# Regulatory networks driving expression of genes critical for glioblastoma are controlled by the transcription factor c-Jun and the pre-existing epigenetic modifications

**DOI:** 10.1186/s13148-023-01446-4

**Published:** 2023-02-27

**Authors:** Adria-Jaume Roura, Paulina Szadkowska, Katarzyna Poleszak, Michal J. Dabrowski, Aleksandra Ellert-Miklaszewska, Kamil Wojnicki, Iwona A. Ciechomska, Karolina Stepniak, Bozena Kaminska, Bartosz Wojtas

**Affiliations:** 1 Laboratory of Molecular Neurobiology, Nencki Institute of Experimental Biology, Warsaw, Poland; 2 Postgraduate School of Molecular Medicine, Medical University of Warsaw, Warsaw, Poland; 3 Institute of Computer Science of the Polish Academy of Sciences, Warsaw, Poland; 4 Laboratory of Sequencing, Nencki Institute of Experimental Biology, ul. Ludwika Pasteura 3, 02-093 Warsaw, Poland

**Keywords:** Glioblastoma, Transcriptional deregulation, Transcription factors, Gene expression, Chromatin accessibility, DNA binding, DNA methylation

## Abstract

**Background:**

Glioblastoma (GBM, WHO grade IV) is an aggressive, primary brain tumor. Despite extensive tumor resection followed by radio- and chemotherapy, life expectancy of GBM patients did not improve over decades. Several studies reported transcription deregulation in GBMs, but regulatory mechanisms driving overexpression of GBM-specific genes remain largely unknown. Transcription in open chromatin regions is directed by transcription factors (TFs) that bind to specific motifs, recruit co-activators/repressors and the transcriptional machinery. Identification of GBM-related TFs-gene regulatory networks may reveal new and targetable mechanisms of gliomagenesis.

**Results:**

We predicted TFs-regulated networks in GBMs in silico and intersected them with putative TF binding sites identified in the accessible chromatin in human glioma cells and GBM patient samples. The Cancer Genome Atlas and Glioma Atlas datasets (DNA methylation, H3K27 acetylation, transcriptomic profiles) were explored to elucidate TFs-gene regulatory networks and effects of the epigenetic background. In contrast to the majority of tumors, c-Jun expression was higher in GBMs than in normal brain and c-Jun binding sites were found in multiple genes overexpressed in GBMs, including *VIM*, *FOSL2* or *UPP1*. Binding of c-Jun to the *VIM* gene promoter was stronger in GBM-derived cells than in cells derived from benign glioma as evidenced by gel shift and supershift assays. Regulatory regions of the majority of c-Jun targets have distinct DNA methylation patterns in GBMs as compared to benign gliomas, suggesting the contribution of DNA methylation to the c-Jun-dependent gene expression.

**Conclusions:**

GBM-specific TFs-gene networks identified in GBMs differ from regulatory pathways attributed to benign brain tumors and imply a decisive role of c-Jun in controlling genes that drive glioma growth and invasion as well as a modulatory role of DNA methylation.

**Supplementary Information:**

The online version contains supplementary material available at 10.1186/s13148-023-01446-4.

## Background

Malignant gliomas account for 80% of malignant central nervous system (CNS) tumors and are the most common primary CNS tumors in adults. An integrated classification introduced by the World Health Organization (WHO) categorized gliomas into malignancy grades (I–IV) based on tumor morphology and molecular information [1, 2]. Comprehensive transcriptomic, genomic and epigenetic analyses showed substantial differences between low-grade gliomas (LGGs, WHO grade I–II) and high-grade gliomas (HGGs, WHO grade III–IV) [2]. Glioblastoma (GBM) is a highly malignant, diffuse WHO grade IV tumor with a poor prognosis. The mean patient survival is 15 months due to a lack of efficient conventional and emerging therapies, including immune checkpoint blockade therapies. Deregulation of transcription due to aberrant activation of signaling pathways is manifested by altered activation, repression and/or temporal/spatial regulation of many genes [3], all of which contribute to tumor initiation and progression. We have recently demonstrated the widespread deregulation of chromatin accessibility, histone modifications and transcriptional profiles in GBMs in comparison with benign gliomas [4].

A crucial step in the regulation of gene expression is an initiation of transcription, which is strictly regulated by DNA-binding proteins known as transcription factors (TFs) [5]. Altered TF activities had been linked to a variety of cancers, with an estimated 20% of oncogenes being identified as TFs [6]. TFs bind mainly in open chromatin regions, to gene promoters and enhancers, and usually cooperate with other DNA-binding proteins to regulate gene expression [7, 8]. Different mechanisms such as gene amplifications, point mutations, expression changes along with DNA methylation or histone modifications can influence TF activities in cancer [9]. Overexpression of certain TFs may serve as a prognostic marker in malignant gliomas [10].

Initial ENCODE studies revealed three main TFs localizing almost exclusively within accessible chromatin: c-Jun, GATA1 and NRF1 [11]. c-Jun is a component of the activator protein-1 (AP-1), a dimer composed of proteins of the Jun (c-Jun, JunB, JunD), and Fos (c-Fos, FosB, FRA-1 and FRA-2) families [12]. The AP-1 complex comprising c-Jun stimulates cell proliferation through the repression of tumor suppressor genes [13] or the induction of *CYCLIN D1* [14–16]*.* JunB and JunD act frequently as negative regulators [17, 18].

To identify TF-gene regulatory networks driving transcriptional deregulation in gliomas, we exploited public TCGA datasets as well as the Glioma Atlas created in our laboratory, which encompasses genome-wide profiles of chromatin accessibility, histone modifications, DNA methylation and gene expression from the same tumor sample [4] (a summary information is presented in Additional file 2: Fig. S1). Integrating and intersecting the acquired data resulted in mapping of active promoters and enhancers in benign and malignant gliomas and led to identification of GBM-specific active sites [4]. In the present study, we combined these multiple datasets with newly acquired data on chromatin accessibility, H3K27 acetylation and gene expression in two cultured human glioma cell lines to predict gene regulatory networks and select candidate TFs enriched in GBMs. We discovered a putative set of TFs, including c-Jun, that control genes involved in the growth and invasion of GBM. We verified the binding and presence of c-Jun at two candidate gene promoters (*VIM, UPP1)* in glioma cells and found a stronger binding in GBM-derived cells in comparison with cells derived from the low grade glioma. We demonstrated that GBM-specific DNA methylation patterns exist in c-Jun target genes and verified that methylation in the c-Jun binding site may affect its binding to the target sequence. Our findings associate specific TF-gene regulatory networks with malignant glioma pathogenesis.

## Results

### Identification of TF binding sites enriched in open chromatin regions in glioblastoma

**Fig. 1:**
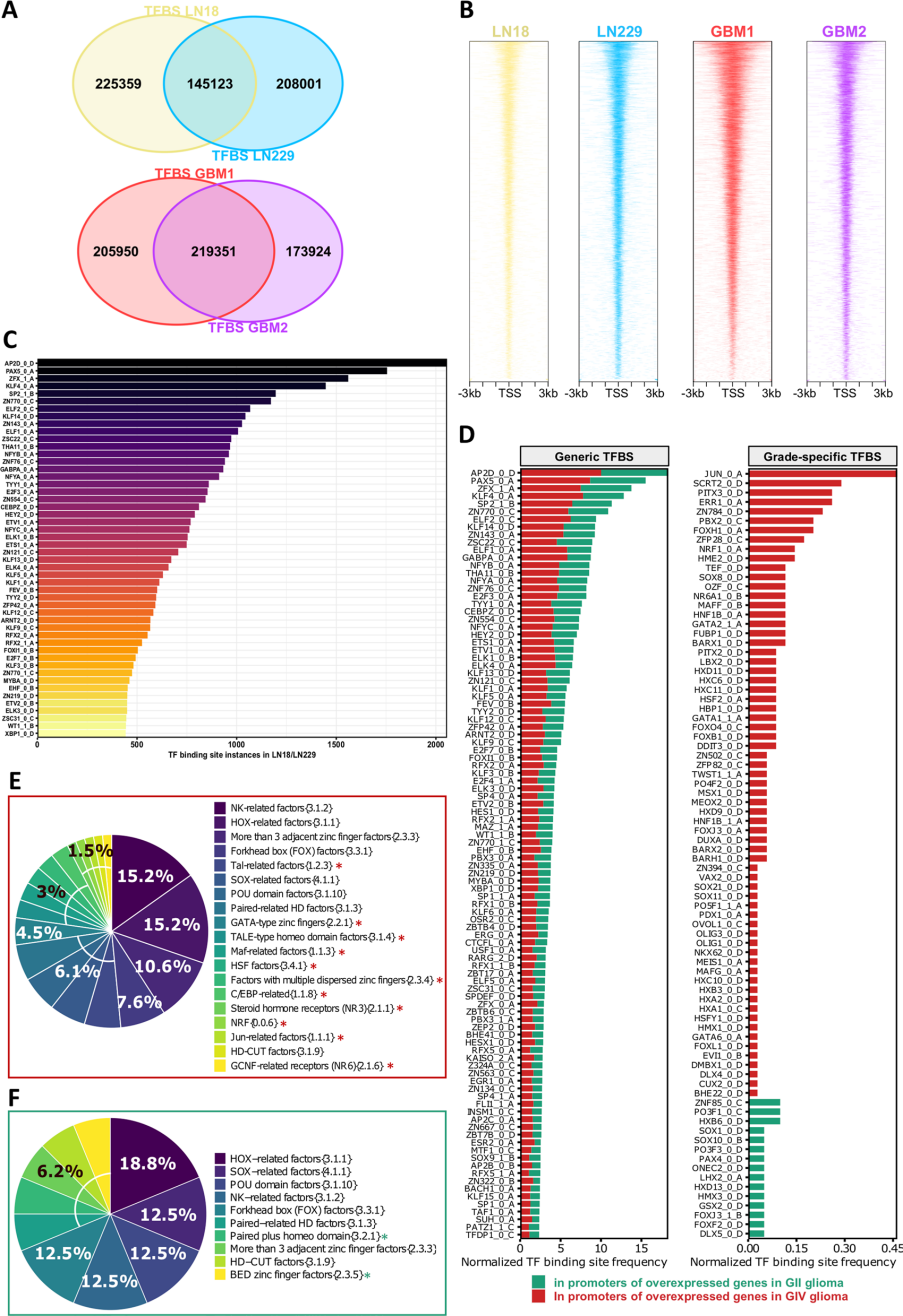
Global characterization of transcription factor binding sites in open chromatin regions in glioblastoma cell lines and glioblastoma specimens. **A** Total number of predicted transcription factor binding sites (TFBS) in open-chromatin regions using ATAC-seq fragments and position-weight matrices (PWMs) motifs in established human glioma cell lines LN18 and LN229 and in glioblastoma samples. In silico TFBS predictions for both cell lines were selected for downstream analysis. **B** Profile heatmap of total ATAC-seq peaks identified around transcription start sites (TSS) in cell lines and GBM specimens. **C** Top 50 occurrences of TFBS (identified with HOmo sapiens COmprehensive MOdel COllection - HOCOMOCO v11) in gene promoters (TSS ± 1.5 kb) in LN18 and LN229 glioma cell lines; the last letter (A–D) represents the quality, where A represents motifs with the highest confidence and the number defines the motif rank, with zero indicating the primary model (primary binding preferences). **D** Prediction of “generic” (left-panel) and “grade-specific” (right-panel) TFBS  in the promoters (TSS ± 1.5 kb) of dysregulated genes in gliomas of grade IV and II. The abscissa represents a normalization factor for TFBS occurrences in which the total number of differentially expressed genes in a given glioma grade is taken into account. **E**, **F** Transcription factor (TF) families (HOCOMOCOv11) with putative binding sites in the promoters of overexpressed genes in IV glioma (**E**) and grade II glioma (**F**). Unique TF families found in either group are highlighted with asterisks

First, we predicted transcription factor binding sites (TFBS) from chromatin accessibility peaks generated by ATAC-seq and identified in LN18 and LN229 human glioma cells. Only peaks consistently detected in both cell lines were considered for further analysis. Secondly, we compared the obtained results with our in-house generated ATAC-seq data from two GBM samples (having sufficiently deep sequencing coverage to reliably detect TFBS) [4]. Data were acquired from a genome browser [19]. At least 60% overlapping TFBS calls were identified in cell lines and tumor samples (Fig. 1A) and only the TFBS detected in both cell lines (145,123) or GBMs (219,352) were further analyzed. Chromatin accessible regions were noticeably different between cultured glioma cells and tumor samples (Fig. 1A), which may reflect the cellular heterogeneity of GBMs. We created heatmaps of all detected peaks to identify the ATAC-seq signal enrichment near the transcription start site (TSS), and evaluated the precise distribution of peaks in these promoters. Open chromatin peaks occurred mostly in the vicinity of the TSS (Fig. 1B).

TFBS identified within open chromatin regions in cultured glioma cells contained numerous motifs for the AP2D, PAX5, and ZFX binding proteins, among many others (Fig. 1C). Next, we performed an analysis of differentially expressed genes (DEG) using TCGA data (Additional file 2: Fig. S1) to identify genes that were either overexpressed in GIV gliomas or in benign gliomas (WHO grade II gliomas, GII). Then, we searched for TFBS only within the promoter regions of these genes (TSS ± 1.5 kb). We found 3454 genes overexpressed in GBMs and 2010 genes overexpressed in GII gliomas (Additional file 2: Fig. S1). TFBS in the promoter regions of these genes were referred to as “generic TFBS” if they were found in any set of overexpressed genes, and “grade-specific TFBS” if they were only identified in one set (Fig. 1D). “Generic TFBS” motifs occurred in equal proportions in the GIV and GII specific gene promoters, indicating that these motifs could be engaged in housekeeping or brain specific functions (Fig. 1D). However, we found 240 TFBS that were present in the promoters of genes overexpressed in GIV gliomas, and this set included binding sites for c-Jun, SCRT2, PITX3, ERR1, or ZN784. TFBS for factors such as ZNF85, PO3F1, HX36, SOX1, and SOX10 were found in genes overexpressed in GII gliomas (Fig. 1D).

We annotated these grade-specific transcription factors into TF families using the HOCOMOCO v11 database [20], and found that specific TF protein families may contribute to transcription regulation specifically in GIV or GII gliomas (Fig. 1E, F). Binding sites for TFs belonging to families such as Tal-related factors, GATA-type zinc fingers, or Jun-related factors were present only in the genes overexpressed in GIV gliomas (Fig. 1E). Motifs for some TF families, such as NK-related factors, HOX-related factors, and POU-related factors, were enriched in both GII and GIV gliomas. These findings suggest that TFs from particular TF families drive gene expression networks deregulated in GIV gliomas.

Next, we examined transcriptomic differences between GIV and GII gliomas from the TCGA dataset. Patient samples clustered depending on a glioma grade (Additional file 2: Fig. S2A) in agreement with previous studies [21–23]. Numerous transcriptomic differences between GIV and GII gliomas were detected (Additional file 2: Fig. S2B). The genes overexpressed in GII gliomas were functionally related to synaptic and neuronal functions (Additional file 2: Fig. S2C, D), whereas the genes overexpressed in GIV gliomas were related to immune responses and cell cycle, as shown by the Gene Set Enrichment Analysis (GSEA). Furthermore, a pathway enrichment analysis revealed that genes up-regulated in GIV were associated with the p53 signaling pathway, cell cycle, IL-17 signaling, nucleosome assembly, and extracellular matrix (ECM) organization, whereas genes up-regulated in GII were associated with neuroactive interactions, GABAergic synapses and synaptic plasticity (Additional file 2: Fig. S2E, F). Enrichment in gene signatures related to neuronal functions may reflect higher resemblance of GII tumors to normal neuronal tissue in contrast to malignant GIV.

### *c-Jun* upregulation in malignant gliomas

**Fig. 2:**
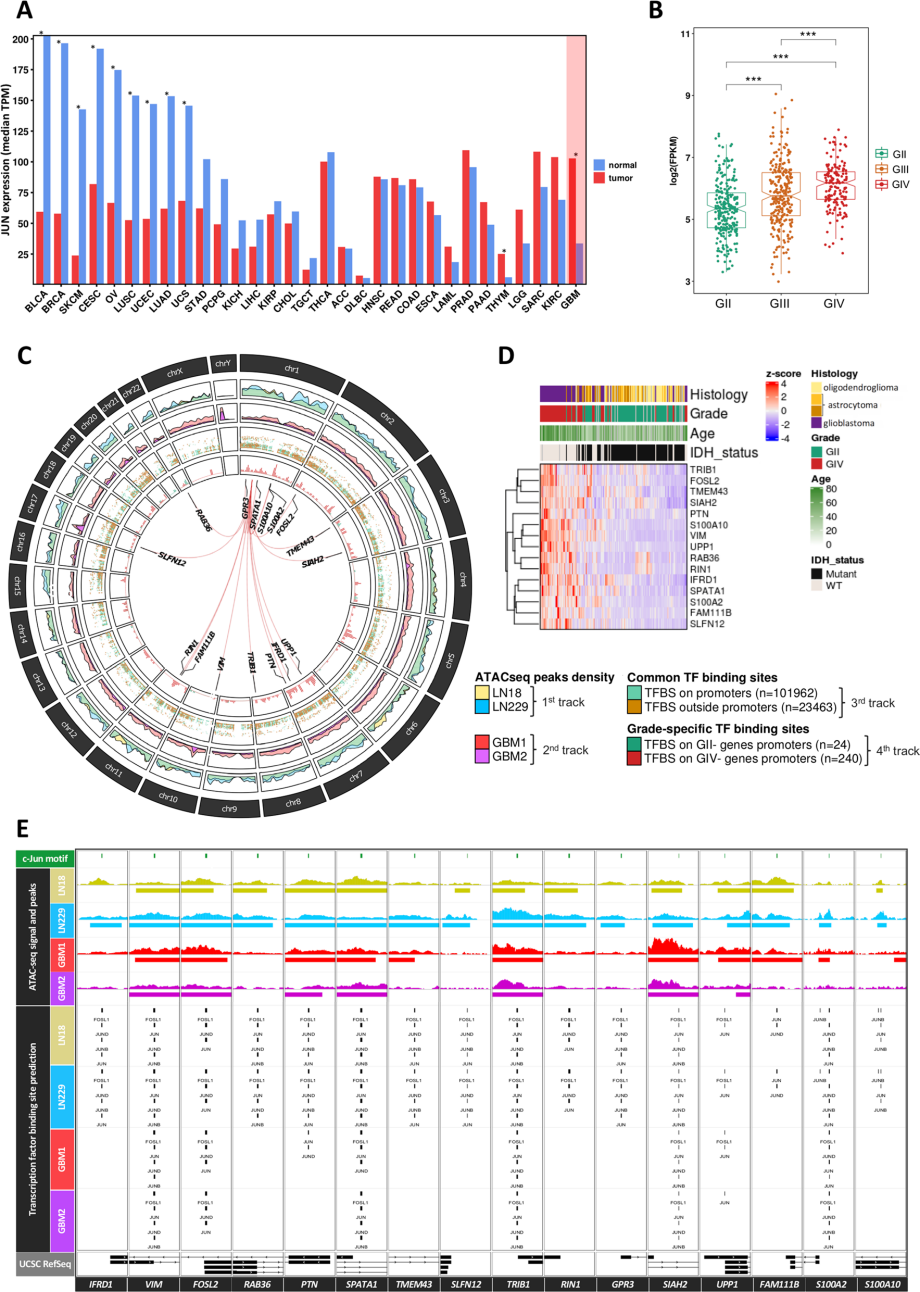
c-Jun dysregulation in the Pan-cancer atlas and the TCGA glioma dataset and identification of genomic targets in open-chromatin regions. **A** *JUN* mRNA expression profile ordered by expression differences between tumor samples (TCGA) and paired normal tissues (TCGA normal + GTEx normal). The differential expression was calculated using one-way ANOVA (Tumors or Normal, *p value < 0.05). **B** *c-Jun *mRNA expression across glioma grades using the TCGA data. The differential expression was calculated using Wilcoxon rank-sum statistical test (*p value < 0.05, **p value < 0.01 and ***p value < 0.001). **C** Chromatin accessibility profiling (1st and 2nd tracks) and TFBS predictions in or outside promoters (3rd track). The 4th track depicts TFBS predictions in overexpressed genes in certain glioma grades, and red lines connect *JUN* gene location (*chr1:58,776,845–58,784,048*) to each of the c-Jun-controlled genes in GBM. **D** Unsupervised hierarchical clustering heatmap of c-Jun gene targets in grade II and grade IV gliomas from the TCGA dataset (248 grade II gliomas; 160 grade IV gliomas). **E** Landscape of c-Jun binding prediction in the cis-regulatory regions of selected overexpressed GIV genes, in the studied cell lines and GBM patient samples. Location of the identified c-Jun motif is shown in green. The ATAC-seq signal and MACS2 broad peaks for each cell line and GBM patient sample are shown separately. Exons (rectangles) and introns (lines) are depicted as well as the gene orientation (arrows) in the UCSC gene composite track

The presented findings show that c-Jun transcription factor is predicted to bind to the promoters of the genes overexpressed in GIV gliomas (Fig. 1D). We used the Pan-Cancer and glioma TCGA datasets to investigate *JUN* expression in various types of cancers and corresponding normal tissues (Fig. 2A). In most cancers (i.e., BLCA, BRCA, SKCM, CESC, OV, LUSC, UCEC, LUAD, and UCS; full names in Methods), *JUN* expression was significantly lower in tumors than in non-tumor tissues. Only in thymoma (THYM) and GBM, *JUN* expression was increased as compared to normal adjacent tissues. Furthermore, the TCGA dataset showed that *JUN* expression increased with glioma grade and was highest in GIV gliomas (Fig. 2B).

### Identification of c-Jun-gene regulatory networks in cultured human glioma cells

Patterns of ATAC-seq peaks, representing chromatin accessible regions, were consistent in LN18 and LN229 glioma cells (Fig. 2C, 1st track) and showed a considerable similarity to the patterns of accessible chromatin identified in GBMs (Fig. 2C, 2nd track). After intersecting ATAC-seq peaks with the H3K27 acetylation peaks, we identified 101,962 TFBS in the promoter regions, accounting for ~ 81.3% of all TFBS predictions; whereas only ~ 18.7% of TFBS were found outside of the promoter regions (Fig. 2C, 3rd track).

In the promoters of genes overexpressed in GII gliomas (Additional file 2: Fig. S1), we detected 24 putative TFBS, while in the promoters of genes overexpressed in GBMs, we found 240 putative TFBS (Fig. 2C, 4th track). Interestingly, many c-Jun binding sites were found in the promoters of genes involved in immune-related signaling (*IFRD1*, *UPP1*, and *SLNF12)*, cell proliferation, migration, invasion (*VIM*, *FOSL2*, *PTN*, *SIAH2*, *S100A2*, *S100A10* and *FAM111B)* and radio-resistance (*TRIB1)*. All of these genes were significantly up-regulated in GIV when compared with GII (Fig. 2D). Several Jun or Fos proteins can bind to the same binding sites as c-Jun within regulatory regions of potential c-Jun targets (Fig. 2E). Similar putative TFBS were identified in glioma cells and GBMs in 50% (8/16) of the gene promoters (Fig. 2E, GBM1 and GBM2 TFBS predictions).

We studied if patient survival is associated with the expression of *JUN* and its target genes in GBM-TCGA (Additional file 2: Fig. S3) and LGG-TCGA samples (Additional file 2: Fig. S4). High expression of c-Jun targets*: FOSL2, GPR3, RIN1* and *UPP1* (Log-rank *p* values < 0.05) was associated with a worse prognosis. Patient survival analysis of the LGG patients showed that high expression of c-Jun targets is associated with a worse prognosis.

### Transcriptomic analysis of grade-specific transcription factors

**Fig. 3:**
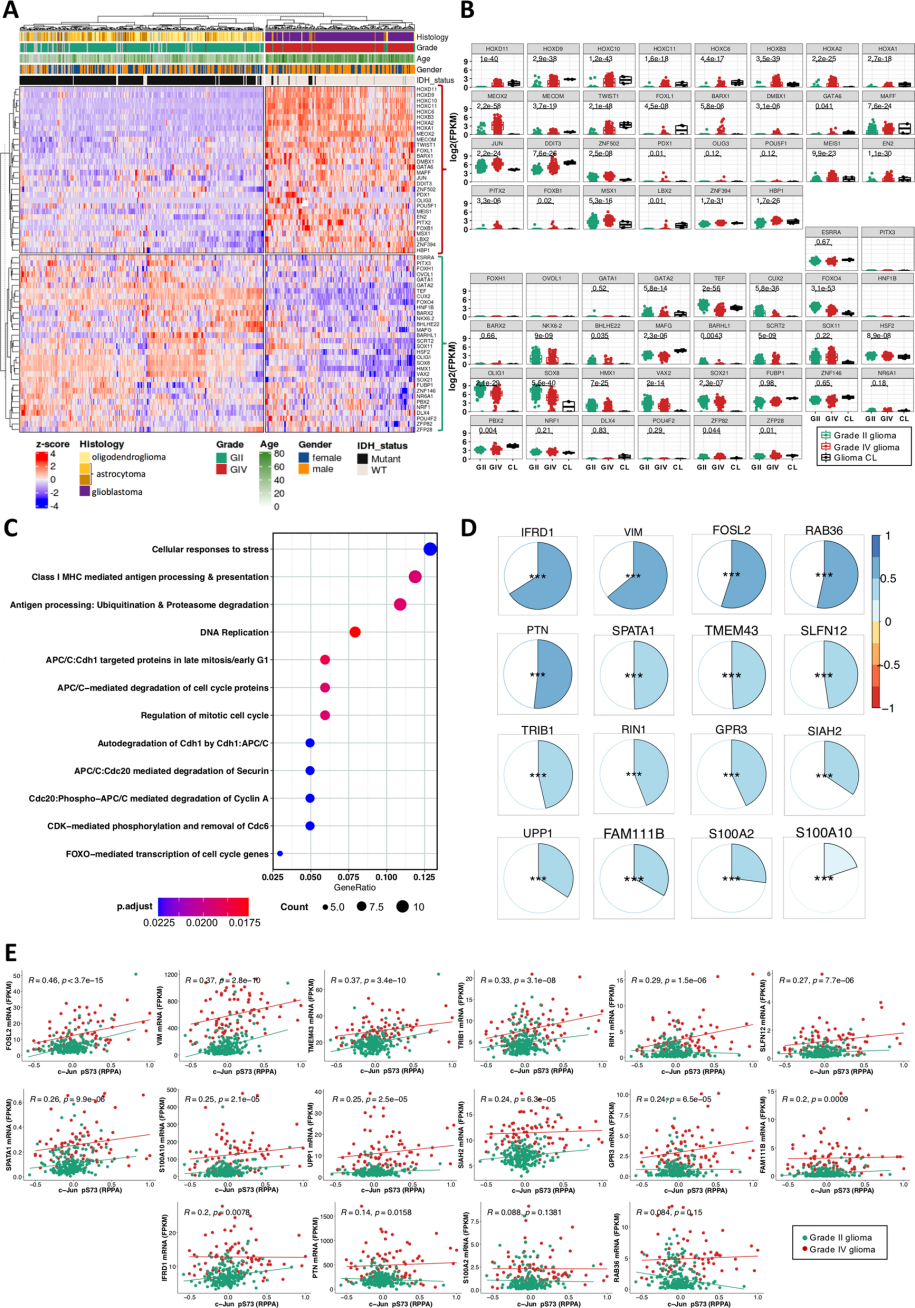
Transcription factor expression differs across glioma grades and c-Jun positively correlates with its target genes. **A** Unsupervised clustering of genes coding for grade-specific transcription factors. The TCGA patients (grade II: 248 patients, grade IV: 160 patients) and genes were clustered using Ward's minimum variance method. Patients who lacked clinical information on Histology, Grade, Age or Gender are illustrated in grey. **B** Normalized transcription factor expression in grade II and grade IV glioma (TCGA RNA-seq data) and in established glioma cell lines LN18 and LN229 (CL; 2 replicates of each shown). TFs were grouped based on dendrogram clusters depicted in A. The adjusted p-values for statistical differences between glioma grades are displayed and the Wilcoxon rank-sum statistical test and Benjamini–Hochberg (BH) correction were used. Transcription factors with a logarithmic expression of zero or nearly zero in glioma patients have no statistical validity. **C** Reactome analysis of genes having grade IV-specific transcription factor motifs in their promoters. BH procedure was used to correct for multiple testing. **D** Correlation of mRNA levels between *JUN* mRNA and its targets (TCGA grade II and grade IV patients). Genes are ordered based on obtained Pearson’s correlation, ranging from blue (coefficient = 1) to red (coefficient =  − 1) and associated *p* values were corrected by multiple testing (*padj < 0.05, **padj < 0.01 and ***padj < 0.001). **E** Pearson's correlation coefficient of c-Jun reverse phase protein array (phosphorylated c-Jun pS73) against mRNA of c-Jun target genes. The adjusted BH p-values for statistically significant correlations are displayed. The data points are color-coded according to the glioma's grade, along with their regression line

The enrichment of TFBS in open-chromatin regions in GIV gliomas (red bars on the right panel in Fig. 1D) suggested that the corresponding TFs regulate genes important for glioma progression. We examined the expression of GIV specific TF encoding genes (64) using hierarchical clustering of TCGA GII and GIV (Fig. 3A). We found that while most of them are highly expressed in GIV, some are more prominent in GII. Many HOMEOBOX (HOX)-related genes (*HOXD11, HOXD9, HOXC10, HOXC11, HOXC6, HOXB3, HOXA2, HOXA1*) were associated with a glioma grade and significantly overexpressed in GIV (Wilcoxon rank-sum and BH padj < 0.05). *HOX* genes are involved in developmental processes [24] so they are not expressed in the adult brain, but they are re-expressed in malignant gliomas and linked to tumorigenesis [25]. To exert gene-specific regulatory outcomes, HOX factors must interact with other TFs (that are expressed in a tissue- and cell type-specific manner) [26]. Expression of *JUN* was significantly up-regulated in GIV when compared to GII (Fig. 3A). Genes coding for TFs upregulated in GIV (Fig. 3B) were statistically significantly overexpressed in 90% of the cases (27/30), whereas genes coding for TFs that were associated with GII (Fig. 3B) were significantly overexpressed across glioma grades in only 53.15% (17/32) cases. Factors such as *MEOX2, TWIST1, MAFF, DDIT3, MEIS1* were overexpressed in GIV. A number of TF coding genes were specifically overexpressed in GII (Fig. 3B).

The expression of preselected TFs was also evaluated in RNA-seq data from human LN18 and LN229 glioma cells (Fig. 3B, black boxplots). Gene expression medians for many genes encoding TFs were consistent with the patterns detected in the tumor samples (Fig. 3B). Genes coding for transcription factors PDX1, OLIG3, POU5F1, PITX3, FOXH1, OVOL1, GATA1, HNF1B, BARX2, POU42F2 were expressed at a very low level (Fig. 3B). Even though their motifs were found in the promoters of GIV-related genes, expression of associated TFs might be lost in cultured cells or they have specific expression in non-tumor cells in GBMs.

Focusing on GIV-specific TFs (Fig. 1D, right panel), we selected 166 genes that have at least one TF motif predicted in the gene promoter and were significantly upregulated in GIV compared to GII (student's *t* test and FDR < 0.05). Then, to better understand biological functions of these genes, we performed a pathway enrichment analysis (Fig. 3C). We found that many of these genes are involved in cellular stress responses, DNA replication, cell cycle, and antigen processing and presentation. This suggests that the identified GIV-specific TFs may influence expression of critical genes involved in glioma progression.

### Expression of *JUN* positively correlates with expression of its putative targets

We hypothesized that high levels of c-Jun will result in the increased expression of its target genes. We calculated the correlation between the *JUN* mRNA level and the expression of c-Jun targets in GII and GIV in the TCGA dataset (Fig. 3D). We found a positive and significant Pearson's correlation (adjusted *p* values < 0.05) in all of the cases, with the highest positive correlation for genes encoding interferon related developmental regulator 1 (*IFRD1*), Vimentin (*VIM*), and FOS Like 2 (*FOSL2*). Using publicly available reverse protein phase assay (RPPA) data [27], we compared the level of the phosphorylated c-Jun (serine 73, S73) and expression of sixteen genes in TCGA glioma samples (Fig. 3E). The higher levels of phosphorylated c-Jun significantly correlated with mRNA levels of putative c-Jun targets. Based on the mRNA-to-mRNA correlation (Fig. 3D) as well as the phosphorylated c-Jun-to-mRNA correlation, *FOSL2* and *VIM* were the most positively correlated targets (Fig. 3E).

### Motifs for c-Jun and other basic leucine zipper proteins are enriched in distal regulatory regions in gliomas

**Fig. 4:**
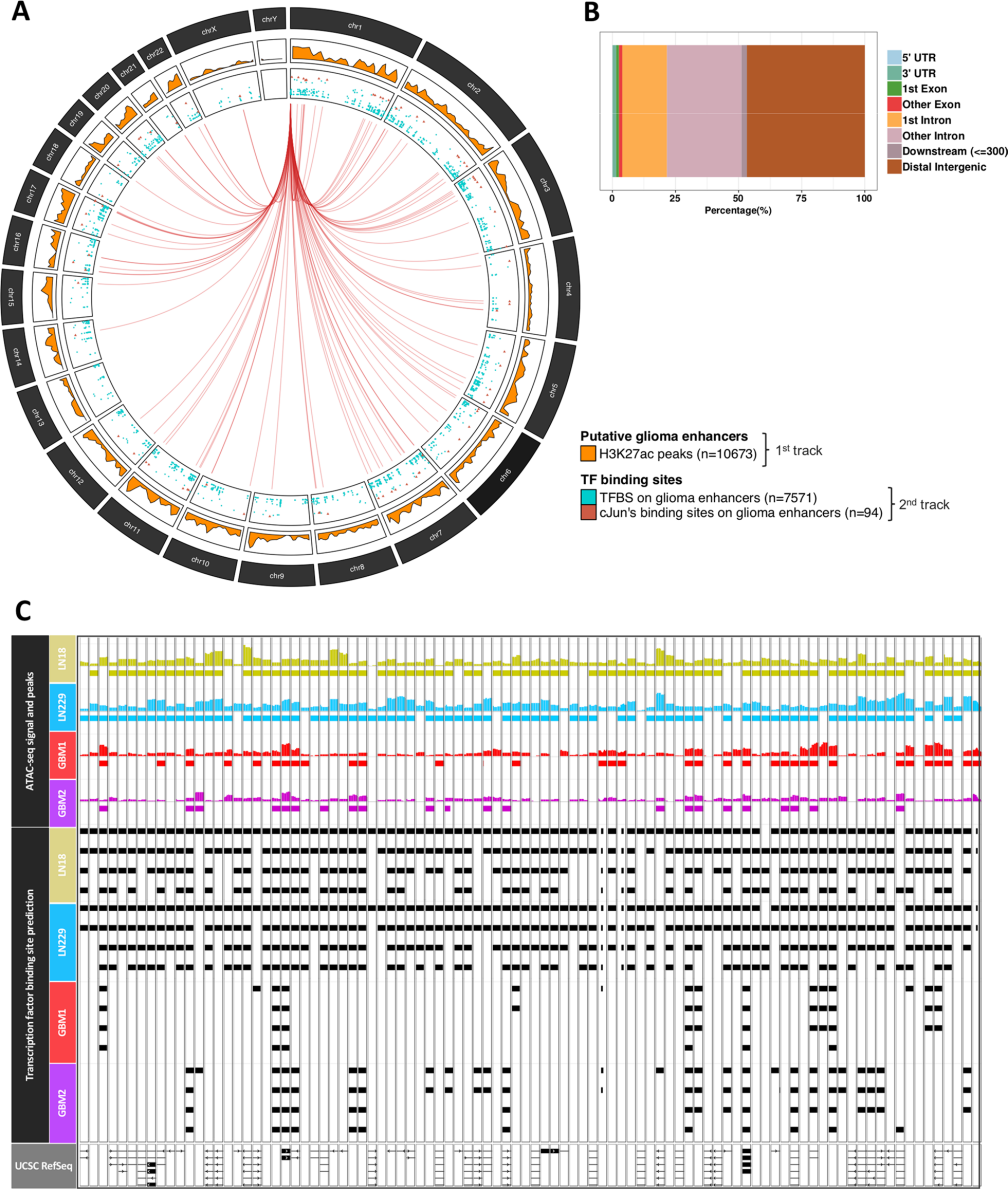
The integration of the glioma enhancer atlas in the context of c-Jun and related factors. **A** The density of the ChIPseq H3K27ac peak from the predicted  glioma enhancer atlas (1st track) identified by Stepniak et al. TFBS motif predictions in LN18 and LN229 glioma cell lines inside enhancers and c-Jun binding sites are displayed separately (2nd track). Each putative JUN motif found in glioma enhancers is linked to *JUN* gene position (*chr1:58,776,845–58,784,048*). **B** Feature distribution of glioma enhancer (H3K27ac peaks). **C** Integration of glioma enhancers and chromatin openness in glioma cell lines and GBM specimens with TFBS for c-Jun and other bZIP proteins. Exons (rectangles) and introns (lines) are depicted as well as the gene orientation (arrows) in the UCSC gene composite track

We had previously identified enhancers enriched in active histone H3K27ac marks in topologically associating domains (TADs) in GI (pilocytic astrocytomas, PAs), and HGGs (diffuse astrocytomas, DAs and GBMs) [4]. In the present analysis, we focused on common active enhancers found in several glioma patients (Fig. 4A, 1st track). Subsequently, we intersected all predicted TF motifs within these regulatory regions, which yielded 7,571 TF motif instances (Fig. 4A, 2nd track). A total of 94 binding sites for the c-Jun were identified within glioma enhancers (Additional file 1: Table S1).

We evaluated cumulative hypergeometric probabilities to quantify the enrichment of particular TFBS within glioma enhancers and discovered that several basic leucine zipper (bZIP) transcription factors, including c-Jun, are found significantly at the top of our TFBS ranking (Table 1, Additional file 1: Table S2). This finding suggests that in gliomas, the bZIP TF class, which comprises the Fos-, Jun- and Maf-related families, is involved in gene regulation via promoters and enhancers. Indeed, consensus H3K27ac peaks in GBMs were primarily observed in distal intergenic regions, followed by intronic regions (Fig. 4B). This finding shows that many intron DNA sequences may contain important elements contributing to aberrant transcription in tumors.

**Table 1:** Top 15 TF binding probabilities in glioma enhancers calculated by the hypergeometric test

TF model	Occurrences in enhancers	Occurrences in the genome	Hypergeometric test (*p* value)	Hypergeometric test (adj. *p* value)	Consensus logo
FOSL1_0_A	128	597	1,20723E−42	7,58142E−40	
FOSL2_0_A	137	726	4,86818E−39	1,52861E−36	
JUNB_0_A	115	544	7,50317E−38	1,57066E−35	
JUND_0_A	123	623	3,12176E−37	4,90116E−35	
FOS_0_A	119	626	1,9905E−34	2,50007E−32	
BACH2_0_A	67	273	1,21946E−26	1,27636E−24	
JUN_0_A	94	524	1,38731E−25	1,24462E−23	
NFE2_0_A	71	344	2,45514E−23	1,92728E−21	
BACH1_0_A	87	494	3,08603E−23	2,15336E−21	
FOSB_0_A	96	585	3,7038E−23	2,32598E−21	
NF2L2_0_A	63	279	4,66643E−23	2,66411E−21	
MAFB_0_B	33	147	9,6426E−13	5,04629E−11	
ZN554_1_D	48	302	7,42587E−12	3,58726E−10	
MAFF_1_B	30	134	1,0909E−11	4,89348E−10	
MAFK_0_A	16	41	8,72968E−11	3,65483E−09	

Obtained *p* values were corrected using the Benjamini–Hochberg method

The ATAC-seq dataset from LN18 and LN229 glioma cells encompasses 94 c-Jun motifs (Table 1, Additional file 1: Table S1). The GBM ATAC-seq dataset did not contain all of these motif occurrences (Fig. 4C, GBM1 and GBM2 TFBS predictions). Comparison of the enhancers (H3K27ac signal) between GBM and DAs revealed only few significantly different regions in GBMs (Additional file 1: Table S3). This suggests that the distribution of activating histone marks in the majority of distal regulatory areas is similar in both DAs and GBMs or that tumor-derived data are too noisy to detect subtle differences between these malignant tumors.

*IDH1/2* (isocitrate dehydrogenase 1/2) mutations globally change DNA methylation patterns in gliomas [2]. As DNA methylation may affect binding of TFs to specific motifs, we evaluated DNA methylation levels within c-Jun motifs and their flanking regions (c-Jun motif +/− 20 bp) in enhancers detected in gliomas with *IDH1* wild type (IDHwt) or mutant (IDHmut) status from the Glioma Atlas [4]. Out of 94 such loci, nine had significantly different DNA methylation levels among glioma groups with the highest median beta values in GII/GIII-IDHmut gliomas (FDR < 0.05). Within the nine loci we assigned short sequences rich in cytosines: C-rich regions highly overlapping c-Jun motifs. DNA methylation levels of C-rich regions differed significantly among glioma groups (Additional file 2: Fig. S5A). Specifically, differences were detected between GII/GIII-IDHwt and GIV with significantly lower DNA methylation levels in GIV (Additional file 2: Fig. S5B). The results suggest that differential DNA methylation at these nine gene loci may affect c-Jun binding to the motifs, changing the activity of the enhancer.

### c-Jun binds to the* VIM* gene promoter in human glioma cells

**Fig. 5:**
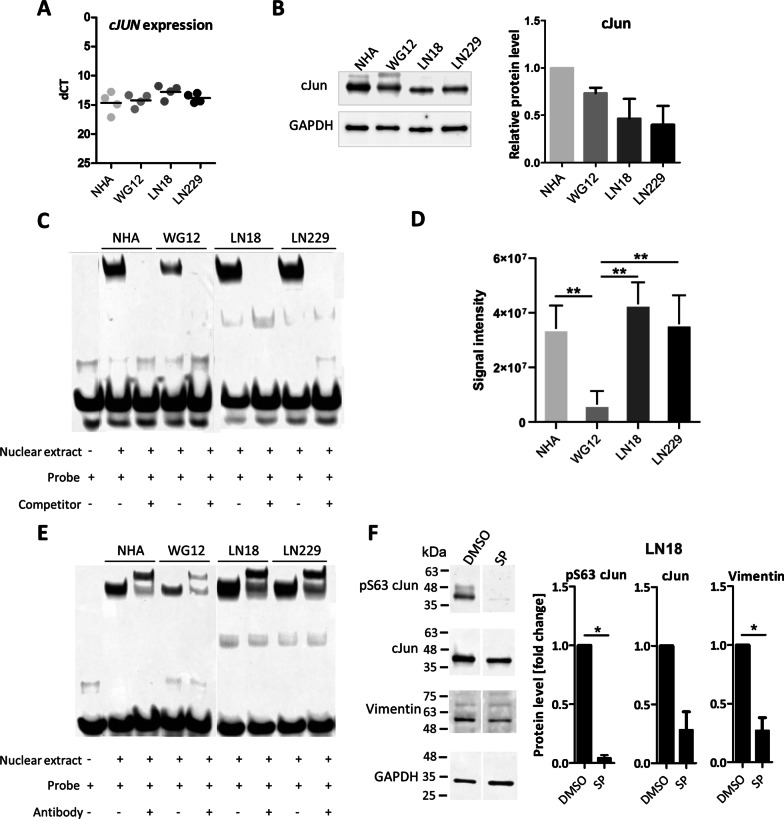
c-Jun transcription factor binds to the *VIM* promoter in human astrocytes and glioma cell lines. c-Jun levels in normal human astrocytes (NHA), low-grade glioma patient-derived cell cultures (WG12) and established glioma cell lines (LN18, LN229). **A** *c-JUN* mRNA expression was evaluated by RT-qPCR. Data were normalized to the expression of *GAPDH* mRNA determined in the same sample, n = 4. **B** Protein levels of c-Jun analyzed by Western blot with the densitometry of immunoblots. Data were normalized to the levels of GAPDH in the same sample, n = 3, mean± SD. The densitometry is presented as relative values to NHA set as 1. *P* values were calculated using GraphPad software and considered significant when *p < 0.05 (One-way ANOVA). **C** DNA-binding activity of double-stranded DNA from the *Vimentin* promoter site. EMSA was performed using the LightShift Chemiluminescent EMSA Kit. Nuclear extracts were isolated from NHA, WG12, LN18 and LN229. Unlabeled competitor probes were added to lanes 3, 5, 7, and 9, *n* = 3. **D** Densitometry analysis of EMSA presented as signal intensity mean ± SD. One-way ANOVA with Dunnett’s post hoc test revealed significant differences at **p < 0.01,  n = 3.  **E** Supershift EMSA assay for measuring c-Jun transcription factor binding to DNA from the *Vimentin *promoter. Antibody against c-Jun was added to samples in lanes 3, 5, 7, and 9 to verify if the observed shift of the probe band in the gel was dependent on c-Jun binding,  n = 3. **F** Impact of inhibition of c-Jun phosphorylation on the level of Vimentin. LN18 cells were treated for 3 h with SP600125 (SP), an inhibitor of JNK kinases. Protein levels were analyzed by Western blot with the densitometry analysis. Data are presented as relative values to control (cells treated with DMSO, set as 1). Data were normalized to the levels of GAPDH in the same sample. *P* values were calculated using GraphPad software and considered significant when *p < 0.05 (*t*-test), *n* = 3, ± SD

The above described results showed that c-Jun might be involved in controlling the expression of several genes, such as *VIM*, that are overexpressed in GBM similarly as *JUN* and contain a specific TFBS in their gene regulatory regions localized within open chromatin. Vimentin is an intermediate filament essential for cell migration, adhesion and cell division, and is frequently upregulated in cancer or metastatic cells [28, 29]. To verify if c-Jun binds to the *VIM* gene promoter, we performed an electrophoretic mobility shift assay (EMSA) using nuclear extracts from normal human astrocytes (NHA) as a control, GII glioma-derived cell cultures (WG12) and two human established cell lines derived from GIV (LN18, LN229). In all tested cells, c-Jun was expressed and there were no significant differences in mRNA or protein levels (Fig. 5A, B). In EMSA experiments, nuclear proteins from glioma cells bound to the fragment of DNA from the *VIM* promoter, producing a clear shift of the labeled probe (Fig. 5C). The strongest binding to the *VIM* promoter was found in extracts from LN18 and LN229 cells (Fig. 5C, D). The intensity of shifted bands of protein-DNA complexes was evaluated by densitometry and quantified (Fig. 5D). Significantly stronger c-Jun binding to the *VIM* promoter was found in LN18 and LN229 cells as compared to WG12 cells. c-Jun binding to the *VIM* promoter was relatively high in NHA, which may be due to proliferation of these cells in cell cultures (Fig. 5C, D). The presence of c-Jun in the DNA–protein complex was confirmed by a supershift assay, where the probe was further shifted after the incubation with anti-c-Jun antibody (Fig. 5E). The reduction of DNA–protein complexes after the addition of the unlabeled probe competing for c-Jun binding confirmed the binding specificity. Additionally, we used SP600125 (SP), an inhibitor of JNK kinases, which phosphorylate and activate c-Jun, and studied the impact of JNK blockade on *VIM* expression. LN18 cells were exposed to 10 µM SP for 3 h and the protein levels were analyzed using Western blotting. The results from three experiments were evaluated by densitometry and quantified (Fig. 5F). SP efficiently decreased the phosphorylated S63 c-Jun levels, which resulted in a significant reduction of VIM levels. The SP treatment has a modest effect on total c-Jun levels (Fig. 5E), most likely due to the inhibition of the positive autoregulatory loop in c-Jun/*JUN* expression [30]. Similar effects of SP treatment leading to decreased phosphorylated S63 c-Jun and Vimentin levels were observed in LN229 cells, although the change was not significant (data not shown). These results confirm that c-Jun regulates the levels of Vimentin in glioma cells.

### DNA methylation at the promoters of c-Jun putative targets differs in low- and high-grade gliomas

**Fig. 6:**
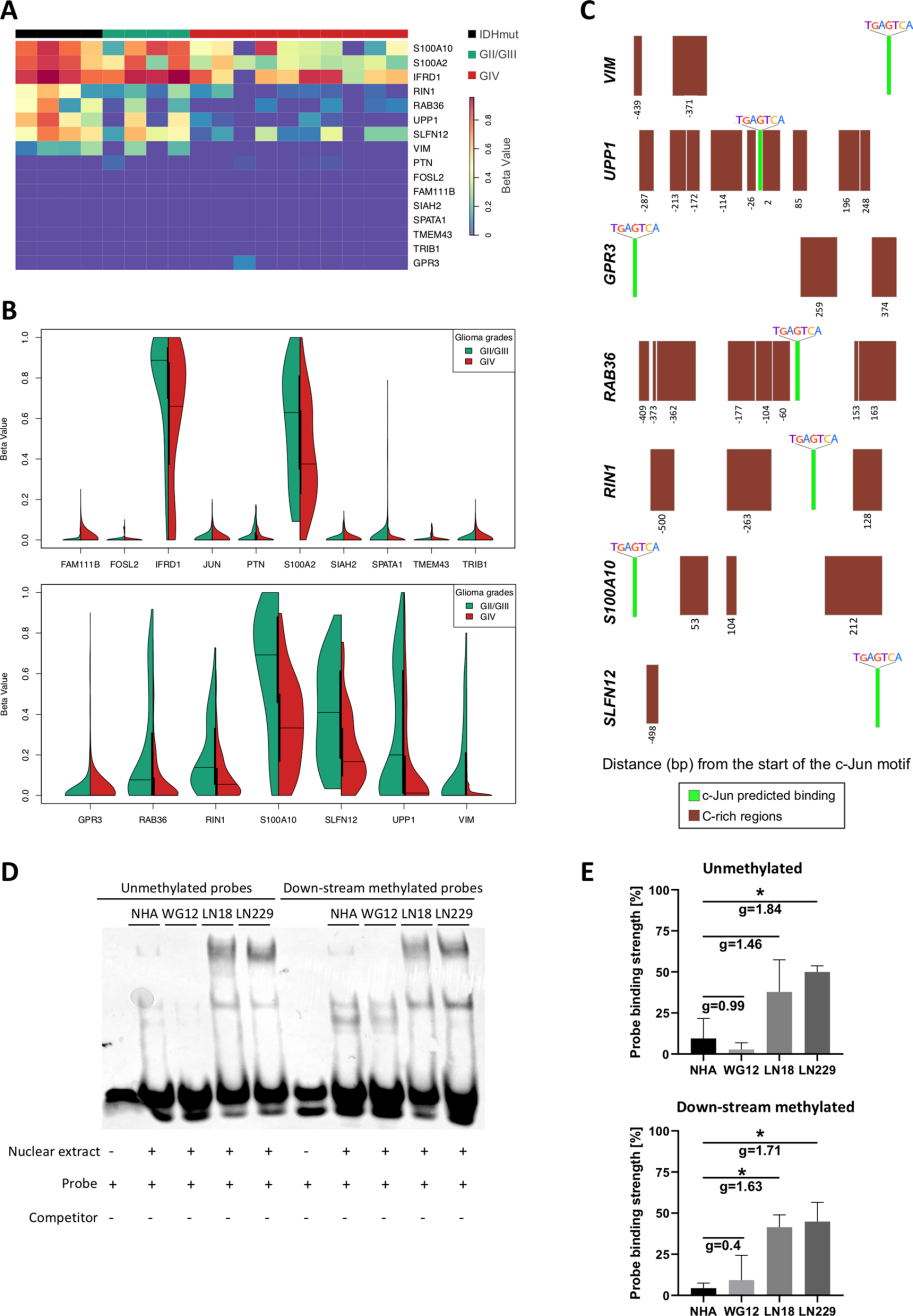
DNA methylation differs in cis-regulatory regions in c-Jun gene targets. **A** Heatmap of hierarchical clustering analysis showing median DNA methylation of promoters of c-Jun target genes (2 kb upstream and 500 bp downstream relative to TSS) in glioma samples. The following labels were used: IDHmut (4 GII/GIII tumors), GII/GIII (4 only GII/GIII-IDHwt tumors) and GIV (10 IDHwt tumors). DNA methylation levels showed as beta values, with 0.0–0.2 representing hypomethylated cytosines and 0.6–1.0 representing hypermethylated cytosines. **B** Differences in beta values distribution in gene promoters with the predicted c-Jun TFBS in GII/GIII-IDHwt versus GIV glioma samples that are statistically non-significant (upper panel) and statistically significant (bottom panel). **C** Distance of c-Jun motif to the beginning of differentially methylated C-rich regions between high- and low-grade gliomas. Green boxes represent a c-Jun predicted binding site, while brown boxes show each C-rich region that was found significantly differently methylated between low- and high-grade glioma samples (Chi-squared test at significance level adjusted *p* < 0.05). **D** Competitive electrophoretic mobility assay (EMSA) was used for measuring binding affinity of nuclear extracts from tumor derived cell lines and NHA, to the methylated and unmethylated double-stranded DNA from the *UPP1* promoter site. Binding of proteins from NHA, WG12, LN18, and LN229 to the methylated and unmethylated probes was evaluated. Lane 1, 6: unlabeled probes; lanes 2–5 and 7–10: protein binding. **E** Probe binding strength in EMSA densitometry analysis is expressed as a percentage of the overall variation across all cell lines. One-way ANOVA with Fisher's LSD post hoc tests revealed significant differences at *p < 0.05 and **p < 0.01; Hedge's effect size (*g*) indicates the strength of the difference of the binding between the studied cell lines (medium effects *g* ≈ 0.5; large effects *g* ≈ 0.8), *n* = 3 ± SD

We analyzed DNA methylation patterns at the *JUN* promoter and putative c-Jun regulated promoters (2 kb upstream/500 bp downstream relative to TSS) in GII/GIII-IDHwt and GIV glioma patients from the Glioma Atlas [4]. Among the genes potentially controlled by c-Jun, we identified two clusters: the first containing genes with high DNA methylation in GII/GIII-IDHmut and GII/GIII-IDHwt gliomas but low in GIV gliomas (*RIN1*, *RAB36*, *UPP1*, *SLFN12* and *VIM*), and the second one encompassing genes with low DNA methylation (beta values ~ 0) regardless of the tumor grade (*PTN*, *FOSL2*, *FAM111B*, *SIAH2*, *SPATA1*, *TMEM43*, *TRIB1* and *GPR3*) (Fig. 6A).

Methylation of the *JUN* promoter was low regardless of grade and IDH status (Fig. 6B), suggesting that its differential expression is not regulated by DNA methylation. Many c-Jun target genes had similar DNA methylation at promoters in GII/GIII and GIV gliomas (Fig. 6B, upper panel). The methylation pattern at some c-Jun target gene promoters clearly differed (Fig. 6B, bottom panel), with low DNA methylation at their regulatory regions in GIV gliomas (Chi-square test for two independent groups, FDR < 0.05). A similar relation was found in other cancers [31]. Methylation of a specific cytosine within a c-Jun motif instance (1 cytosine in the c-Jun motif, “dvTGAGTCAYh”, HOCOMOCO version 11) within the promoters of putative c-Jun targets was found. In several cases, CpG methylation in the flanking regions of predicted c-Jun binding sites varied in gliomas of different WHO grades (Fig. 6C, brown boxes). These results suggest that DNA methylation could control expression of c-Jun regulated genes by affecting TF binding to the regulatory DNA regions.

To validate our previous findings, we explored the TCGA dataset [27]. While the Glioma Atlas and TCGA datasets had different cytosine coverage, the latter contains more samples and is an independent dataset. We found that the IDHmut phenotype creates the hypermethylated pattern in the majority of the gene promoters (Additional file 2: Fig. S5C). Moreover, many genes (*PTN*, *SIAH2*, *FAM111B*, *TMEM43*, *FOSL2*, *TRIB1* and *SPATA1*) were hypomethylated regardless of tumor grade (Additional file 2: Fig. S5C). When GII/GIII-IDHmut gliomas were compared to GIV gliomas, significant differences of DNA methylation in the promoters of *S100A2, RAB36, RIN1, UPP1,* and *VIM* were found (Additional file 2: Fig. S5C). Finally, when DNA methylation and transcriptomic data were correlated, DNA methylation was negatively related to gene expression in the majority of cases (Additional file 2: Fig. S5D). The results suggest that methylation of some c-Jun target genes might have an impact on c-Jun binding and transcription of those genes.

### c-Jun binding to the *UPP1* gene promoter is unaffected by DNA methylation

DNA methylation can repress transcription by preventing specific TFs binding to their recognition motifs, while removing site-specific DNA methylation might reverse the process [32, 33]. In gliomas, DNA methylation is tightly associated with mutations in genes coding for the IDH1/2 which results in glioma CpG island methylator phenotype (G-CIMP) [34, 35]. Patients with G-CIMP + tumors have a better prognosis [36]. Interestingly, specific CpG loci in genes of the homeobox family (*HOXD8, HOXD13 and HOXC4*), among other regulators, are differentially methylated between patients with short- and long-term survival [36].

We explored the previously collected data on DNA methylation in gliomas of different grades [4]. The analysis of DNA methylation profiles in the promoters of GIV-specific genes with c-Jun binding sites showed different levels of methylated cytosines within those promoters (Fig. 6A). Furthermore, some proximal c-Jun binding sites were differently methylated in C-rich regions (Fig. 6C). In GII/GIII-IDHwt gliomas, the proximal areas to c-Jun binding sites (− 26 bp and + 2 bp) exhibited highly methylated cytosines in comparison with similar sites in GBMs (Fig. 6C). In particular, the *UPP1* gene promoter contained numerous differentially methylated cytosines around the c-Jun binding site.

To study the effects of DNA methylation on c-Jun binding, we determined binding of the nuclear extracts from NHA, WG12, LN18 and LN229 glioma cells to unmethylated and methylated *UPP1* promoter region using EMSA. Nuclear extracts from LN18 and LN229 produced a shift of the *UPP1* probes and the binding of nuclear extracts from WG12 cells to both probes was lower (Fig. 6D). There was no difference in binding unmethylated/methylated probes in any of the samples (Fig. 6D, E). Only in NHA, methylation of the probes in the flanking regions of the *UPP1* gene promoter had a minor effect on the formation of DNA–protein complexes (Additional file 2: Fig. S6).

## Discussion

In this study, we analyzed a broad range of publicly available datasets and experimental data from gliomas and human glioma cultured cells to identify novel TF-gene regulatory networks contributing to transcriptional deregulation in malignant brain tumors. Using chromatin accessibility data from glioma samples and cultured glioma cells, we identified GBM-specific TFs binding sites that were also present in human LN18 and LN229 glioma cells. ATAC-seq was used to identify accessible chromatin regions, the majority of which were localized at gene promoters. Open chromatin areas were enriched in TFBS encompassing binding sites for AP2D, PAX5, and ZFX transcription factors. The activator protein 2 (AP-2) is involved in a variety of pathological processes, including cancer [37]. Transcription factor PAX5 (*Paired Box Gene 5*) promotes gliomagenesis and cooperates with factors such as MYC, FOS, or JUN, which are highly expressed in gliomas [38]. The zinc finger and X-linked transcription factor (ZFX) is associated with proliferation, tumorigenesis, and poor patient survival in a variety of human cancers [39], and maintains self-renewal and tumorigenic potential of glioma stem like cells by upregulating *c-Myc* expression [40]. While the available data do not allow to solve a “chicken-egg” dilemma, it is tempting to speculate that increased chromatin accessibility in GBMs and the enrichment of specific TFBS in the promoters of cancer-related genes, result in the establishment of a novel TF-gene regulatory network driving tumorigenesis.

A close inspection of genes overexpressed in GBMs versus LGG revealed the enrichment in specific TFBS in the promoters of these genes, which suggests the contribution of the TFs to glioma development or progression. While, it is clear that grade II astrocytomas and oligodendrogliomas are very different from GBMs in terms of their molecular/genetic profiles, grade II gliomas are benign brain tumors and were selected as a reference to analyze GBM-specific TF networks. As a result of multilayered in silico analyses we identified c-Jun as a prominent candidate for novel TF-gene regulatory network driving glioma growth and invasion. It is a well-known proto-oncogene, involved in proliferation, angiogenesis, migration and apoptosis in several cancers [41, 42]. Interestingly, in the majority of human cancers (bladder, breast, skin, cervical/endocervical, ovarian, lung and uterus), *JUN* expression was higher in normal tissues than in tumors. Only in thymomas and GBMs, *JUN* was significantly overexpressed in tumors when compared to adjacent normal tissue. Moreover, GIII-GIV gliomas showed increased *JUN* expression.

We detected c-Jun binding motifs in the promoters of 16 genes overexpressed in GBMs. Many of these genes (Fig. 2C–E), such as *VIM*, *FOSL2, PTN, GPR3, SIAH2, UPP1, S100A2* are associated with cell migration and epithelial-mesenchymal transition (EMT) in several cancers [43–50]. The positive correlation between c-Jun mRNA/protein and target gene expression indicates that c-Jun most likely regulates the predicted targets in GBMs. All these genes accurately predicted patient survival in TCGA-LGGs but not TCGA-GBMs. One explanation for the observed lack of association in GBMs could be that the expression of these genes is very high in most GBMs, making the distinction between low-expressing and high-expressing patients difficult.

Our observation that several TFBS for Jun- (JunB, JunD) and Fos-related factors (c-Fos, FRA1, FRA2, and FosB) overlap with c-Jun binding sites emphasizes the complexity of c-Jun-regulated networks. c-Jun, a component of the AP-1 complex, may interact with different Jun and Fos proteins to form homo- or heterodimers [51] at the specific promoters and differentially regulate target gene expression. Selection of a dimerizing partner in the AP-1 complex influences which TFBS are recognized and how they are regulated, as some JUN family members lack transactivating domains [52]. The enrichment in c-Jun binding motifs was also identified in glioma enhancers with the H3K27ac activating histone marks, supporting the role of c-Jun in driving GBM specific expression. Expression of genes coding for GBM-specific TFs was higher in malignant gliomas as is exemplified by the expression of *HOX* genes, *JUN* and *TWIST*, in an agreement with previous reports [53, 54].

The abundance of Vimentin*,* measured by immunohistochemistry, is a poor prognostic factor in GBMs [28]. We predicted that c-Jun drives high *VIM* expression in GBMs. The prediction was verified in cultured human GBM cells, in which c-Jun is present and binds to the *VIM* gene promoter, as demonstrated by EMSA and supershift assays. DNA–protein complexes with *VIM* gene promoter probe were more efficiently formed in nuclear extracts from GBM-derived than in benign GII glioma cells. The concentrations of nuclear proteins used for reactions were the same across all cell lines with equivalent levels of c-Jun, and the reduced binding suggests that transcription of c-Jun target genes is lower in GII-derived cells. Regulation of Vimentin by c-Jun in glioma cells was further confirmed with the use of a JNK inhibitor, which decreases phosphorylated c-Jun levels and subsequently Vimentin levels. This is consistent with previous findings in epithelial cells [55], indicating that c-Jun plays a widespread role in oncogenic processes in various cancers.

The physical access of TFs to DNA can be modulated by nucleosome positioning, histone modifications or DNA methylation [33, 56]. When we examined DNA methylation levels at the promoters of c-Jun and its targets in GII/GIII and GIV gliomas, we found differences in DNA methylation in putative c-Jun target genes *S100A10*, *S100A2*, *IFRD1*, *RIN1*, *RAB36*, *UPP1*, *SLFN12* and *VIM*. DNA methylation patterns varied primarily in the flanking regions of the c-Jun binding sites. We found stronger c-Jun binding to the *UPP1* gene promoter in GBM-derived cells than in GII-derived cells using EMSA, although its binding did not depend on the methylation status of the promoter probes. These results indicate that hypermethylated flanking regions do not always have an impact on c-Jun binding to a specific TFBS.

## Conclusion

Identification of TF-gene regulatory networks responsible for the dysregulated expression of certain genes in malignant gliomas holds a key to potential pharmacological manipulations. We demonstrate the increased expression of *JUN* in GBMs (which is uncommon in other pan-cancer samples) and the enrichment of c-Jun binding motifs in the accessible chromatin regions of genes upregulated in GBM samples (GBM-specific genes). Several of these GBM-specific genes are known to be implicated in tumor growth and invasion, and the high expression of putative c-Jun targets is associated with poor survival of glioma patients. We demonstrated that binding of c-Jun to *VIM* and *UPP1* promoters is stronger in human glioma cells derived from GIV versus GII tumors. The inhibition of phosphorylation of c-Jun with the upstream kinase inhibitor resulted in the reduction of Vimentin levels, indicating the role of c-Jun in the regulation of this protein.

## Methods

### Sample collection and human glioma cell cultures

Human established glioma LN18 and LN229 cells were obtained from the American Type Culture Collection (ATCC, Manassas, VA, USA). The cells were cultured in Dulbecco’s Modified Eagle Medium (DMEM) supplemented with 10% fetal bovine serum (FBS, ThermoFisher Scientific), 100 units/mL penicillin and 100 μg/mL streptomycin. In some experiments LN18/LN229 were treated with 10 µM SP600125 inhibitor (Biotechne, cat. no 1496/10) for 3 or 24 h, control cells were treated with 0.05% DMSO. Freshly resected glioma specimens were acquired from two neurosurgical departments of the Medical University of Warsaw and the Mazovian Brodno Hospital. The tissue collection protocol was approved as described [57]. Tumor samples (GBM IDHwt) were transported in DMEM/F-12 medium on ice and processed immediately after surgical resection. Tumor samples were transferred to cold PBS, minced with sterile scissors or a scalpel on a Petri dish kept on ice, and then homogenized with a chilled manual glass mince [57]. Each patient signed a written consent form for the use of tumor tissues, and the samples were then anonymized. Human GBM patient-derived glioma primary cultures WG12 were generated and cultured as described [58] in DMEM/Nutrient Mixture F-12, GlutaMAX™ medium, supplemented with 10% FBS (Gibco Life Technologies, Rockville, MD, USA) and antibiotics. See Additional file 2: Fig. S7 for WG12 cell line's detailed characterization. Normal human astrocytes (NHA cat # CC-2565, Lonza Walkersville, MD, USA) were cultured in a commercial Astrocyte Growth Medium (AGM™ cat # CC-3186, Lonza Walkersville, MD, USA) supplemented with 3% FBS, 1% L-glutamine, 0.1% ascorbic acid, 0.1% human EGF, 0.1% gentamicin, and 0.0025% recombinant human insulin. NTERA-2 cl.D1 (cat # CRL-1973 ™) were purchased from ATCC (Manassas, VA, USA) and cultured in DMEM with GlutaMax-1 and supplemented with 10% FBS. The L0125, and L0627 GBM GSCs (glioma stem-like cells) were provided by Dr Rossella Galli (San Raffaele Scientific Institute, Milan, Italy) [59–61]. Spheres L0125 and L0627 were expanded in vitro in serum-free medium supplemented with 2% B27, 20 ng/ml rh EGF and 20 ng/ml rh bFGF as described before [62, 63]. For differentiation experiments, spheres were triturated for a single cell suspension and seeded onto laminin-coated plates in the medium without cytokines (rh EGF and rh bFGF) but containing 2% FBS and were incubated for 7 days [62]. All cell cultures were grown in a humidified atmosphere of CO2/air (5%/95%) at 37 °C.

### DNA isolation

Genomic DNA was extracted from 50 to 100 mg of glioma sample (depending on the size of the original tumor specimen) and from 2 × 10^7^ LN18 and LN229 cells using Tri-Reagent (Sigma-Aldrich, Munich, Germany). DNA purity was estimated using NanoDrop 2000 (Thermo Scientific, NanoDrop products, Wilmington, USA) and quantity with Agilent Bioanalyzer as described [4].

### ATAC-sequencing

Tumor sample aliquots corresponding to 50–100 mg of tissue were drawn through a syringe needle approximately 50 times. Mechanical homogenization was followed by 5 min of centrifugation at 2400 g at 4 °C. Each pellet was resuspended in 10 ml of cold lysis buffer L1 (50 mM HEPES KOH, pH 7.5, 140 mM NaCl, 1 mM EDTA pH 8.0, 10% glycerol, 5% NP-40, 0.25% Triton X-100, proteinase inhibitor cocktail) and shaken for 20 min at 4 °C. The tissue was then mechanically disrupted, residual debris pre-cleared by filtration through nylon mesh filters, and the lysis buffer was replaced with PBS. Cells were automatically counted using the NucleoCounter NC-100, and 50,000 cells were lysed as previously described [4]. The extracts were filtered using Zymo DNA Clean and Concentrator 5 columns. ATAC-seq library preparation was carried out as described [4]. Finally, ATAC-seq libraries were visualized on a Bioanalyzer 2100 (Agilent Technologies, Santa Clara, CA) and DNA concentration was estimated. Libraries were run in the Rapid Run flow cell and paired-end sequenced (2 × 76 bp) with the HiSeq 1500 (Illumina, San Diego, CA 92122 USA).

### Processing of ATAC-sequencing data

Only two GBM samples had sequence data that were deep enough to support confident motif analysis with the BMO tool. The FastQC tool was used to evaluate the quality of raw fastq data [64]. After trimming ATAC-seq reads with the FASTQ trimmer [65], only reads with a quality of 10 or higher were considered. Reads with incorrect pairing and a length of less than 20 bp were discarded. Using the default parameters, the Bowtie2 aligner [66] was used to map the reads to the human genome (hg38). Only high-quality reads (MAPQ > 30), correctly paired read mates, and uniquely mapped reads were considered for downstream analysis. PicardTools [67] was also used to find and eliminate PCR duplicates. The following parameters in MACS2 were used to center a 200 bp window on the Tn5 binding site (5' ends of reads represent the cut sites), which is more accurate for ATAC-seq peaks: --*broad --nomodel --shift -100 --extsize 200*. Resulting peaks were then intersected with human (hg38) ENCODE blacklisted genomic regions (https://github.com/Boyle-Lab/Blacklist/tree/master/lists) to eliminate anomalous and unstructured signals from the sequencing.

### Selection of genes differentially expressed in gliomas of different WHO grades

We used TCGA data to find overexpressed genes in glioblastomas (GIV) compared to benign gliomas (GII, WHO grade II) using normalized RNA-seq expression values (Fragments Per Kilobase of transcript per Million mapped reads, FPKM). RNA-seq data from 408 glioma patients (248 GII gliomas and 160 GIV gliomas) were analyzed. The biotype of genes from RNA-seq data was restricted to protein-coding genes [68]. We randomly sampled 20 GII and 20 GIV patients from the normalized count matrix (*n* = 200 times) to maximize statistical power and robustness of the gene selection. The sample function from the base R library (version 3.6.2) was used as a sampling technique, with each sample having an equal chance of being chosen.

We then calculated Student's *t* test *p* values for all the genes for each of 200 random comparisons of 20 GII vs 20 GIV, the *p* values were then corrected using multiple testing (FDR), and the means of these adjusted-*p* values from all of these comparisons were calculated. We used DESeq2 methods [69] on the same TCGA dataset to find changes in gene expression and determined the directionality of the change using the log fold change (logFC) criteria. Overexpressed genes in GBM were those that changed significantly based on FDR and had a positive logFC. To investigate the biological significance of our gene selection, we performed pathway enrichment analysis with the ClusterProfiler [70] R library using the Gene Ontology (Biological Processes) and KEGG databases. Here, only genes that differed significantly between glioma grades (GIV vs. GII) were retained (adjusted *p* value means < 0.01).

### Prediction of transcription factor binding from ATAC-seq data

The human HOCOMOCO v11 motif database [20] in the MEME motif format was used to find TFs that could potentially bind to promoters within open chromatin regions. Using the FIMO tool [71], position weight matrices (PWMs) were used to scan the FASTA file of the human genome (hg38). The background nucleotide frequency from hg38 was used and all motif occurrences with a *p* value less than 1e^−4^ on both DNA strands were considered. Motifs found on the mitochondrial genome were discarded for the subsequent analysis. Overall, motif occurrences were computed independently for each of the 735 motifs. The BMO algorithm [72] was used to classify TF binding in human glioma cells and human glioblastoma samples. For further analysis, only motif instances expected to be bound with adjusted *p* values below 0.05 (Benjamini–Yekutieli correction procedure) were used. Only motif instances at the same chromosomal localization in LN18 and LN229 cells were considered. We intersected the resulting TFBS with the promoters of protein coding genes after selecting motif instances that were common in both glioma cell lines. Transcription start sites and their flanking DNA regions upstream (1.5 kb) and downstream (1.5 kb) were used to identify gene promoters [68]. If a particular TF was predicted to bind twice in a single promoter, it was counted as one TFBS because we only considered one TFBS per promoter. By focusing on the top TFs, we could determine the importance of a specific TF and its relationship to gene dysregulation. Finally, the BMO results from two samples of human glioblastoma were compared to the TFBS found in the LN18 and LN229 cells.

### RNA isolation from human glioma cells and RNA-seq processing

Total RNA from glioma cells was isolated using a Qiagen RNeasy kit. In brief, 1 × 10^6^ cells were lysed with 350 µL RLT buffer supplemented with 1% β-mercaptoethanol. The extraction procedure was carried out in accordance with the manufacturer's instructions. The total RNA was eluted with 25 µl of sterile H_2_O and its concentration was estimated with Nanodrop 2000 (Thermo Scientific, NanoDrop products, Wilmington, USA). The KAPA Stranded mRNA Sample Preparation Kit was used to prepare polyA-enriched RNA libraries (Kapa Biosystems, Wilmington, MA, USA). Trimmomatic [73] (version 0.36) with default parameters was used for the transcriptomic analysis to remove Illumina adapters and low-quality reads. Then, RNA sequencing reads were aligned to a reference genome sequence (hg38) with the twopassMode Basic choice enabled in STAR aligner [74] (version 2.6) and all other parameters were set to default. Only properly oriented pairs of reads were considered for downstream analysis. Flag read duplicates and optical duplication estimation was done using MarkDuplicates from Picard Tools [67] (version 2.17.1). RNA-seq mapped reads in paired and reverse stranded mode were summarized and counted by genes using featureCounts software [75] (version 1.5.3). Only genes that were uniquely mapped and had MAPQ mapping quality values of 255 were considered. Raw counts from featureCounts were converted to FPKM values, and genes encoding various transcription factors were selected for further investigation.

### Gene expression profiling in pan-cancer and paired normal tissues

We used the Gene Expression Profiling Interactive Analysis (GEPIA2) [76] to determine *JUN* expression in various human cancers, including in brain tumors. Transcripts per million (TPM) values were extracted from different TCGA and Genotype-Tissue Expression (GTEx) datasets, and median gene expression in each cancer and paired normal tissues was calculated and used as an input to R. The following cancers and corresponding healthy tissues were examined: Adrenocortical carcinoma (ACC) and Adrenal Gland; Bladder Urothelial Carcinoma (BCLA) and bladder; Breast invasive carcinoma (BRCA) and breast; Cervical squamous cell carcinoma and endocervical adenocarcinoma (CESC) and cervix uteri; Colon adenocarcinoma (COAD) and colon; Lymphoid Neoplasm Diffuse Large B-cell Lymphoma (DLBC) and blood, Esophageal carcinoma (ESCA) and esophagus; Glioblastoma multiforme (GBM) and brain; Kidney Chromophobe (KICH) and kidney; Kidney renal clear cell carcinoma (KIRC) and kidney; Kidney renal papillary cell carcinoma (KIRP) and kidney; Acute Myeloid Leukemia (LAML) and bone marrow; Brain Lower Grade Glioma (LGG) and brain; Liver hepatocellular carcinoma (LIHC) and liver; Lung adenocarcinoma (LUAD) and lung; Lung squamous cell carcinoma (LUSC) and lung; Ovarian serous cystadenocarcinoma (OV) and ovary; Pancreatic adenocarcinoma (PAAD) and pancreas; Prostate adenocarcinoma (PRAD) Prostate, Rectum adenocarcinoma (READ) and colon; Skin Cutaneous Melanoma (SKCM) and skin; Stomach adenocarcinoma (STAD) and stomach; Testicular Germ Cell Tumors (TGCT) and testis; Thyroid carcinoma (THCA) and thyroid; Thymoma (THYM) and blood; Uterine Corpus Endometrial Carcinoma (UCEC) and uterus; Uterine Carcinosarcoma (UCS) and uterus. The *JUN* mRNA expression profile was compared between tumor samples (TCGA) and paired normal tissues (TCGA normal + GTEx normal), and statistical significance was determined using one-way ANOVA and disease state (Tumor versus healthy tissue of tumor origin).

### ChIP-sequencing

The QIAseq Ultra Low Input Library Kit was used to create DNA libraries for chromatin immunoprecipitation with the appropriate antibodies (QIAGEN, Hilden, Germany), as described [4]. End-repair DNA was used, adenosines were added to the 3′ ends of dsDNA to create “sticky-ends”, and adapters (NEB, Ipswich, MA, USA) were ligated. Following adapter ligation, uracil was digested in an adapter loop structure by USER enzyme from NEB (Ipswich, MA, USA). Using NEB starters, adapters containing DNA fragments were amplified by PCR (Ipswich MA, USA). The Agilent 2100 Bioanalyzer with the Agilent DNA High Sensitivity chip was used to evaluate the library's quality (Agilent Technologies, Ltd.) To quantify and evaluate the obtained samples, the Nanodrop spectrophotometer (Thermo Scientific, NanoDrop products, Wilmington, USA), Quantus fluorometer (Promega Corporation, Madison, USA), and 2100 Bioanalyzer were used to quantify and evaluate the obtained samples (Agilent Technologies, Santa Clara, USA). The average library size was 300 bp. Libraries were run in the rapid run flow cell and were single-end sequenced (65 bp) in the rapid run flow cell on HiSeq 1500 (Illumina, San Diego, CA 92122 USA).

### Comparison of H3K27ac histone modification across glioma grades

We had acquired histone ChIP-seq data from gliomas of different grades from the Glioma Atlas [4] focusing on activated enhancers from eight diffuse astrocytomas (DAs) and ten GBMs. We used the DESeq2 method [69] to identify H3K27ac ChIP-seq signal differences within enhancer peaks to better capture the differences in active enhancer marks between glioma grades. First, we filtered out peaks found in only one tumor sample and the resulting peakset was used to count single-end reads from BAM files using the featureCounts [75] tool. H3K27ac signal differences between GBMs and DAs were identified, and only regions with adjusted p-values 0.05 were considered as statistically significant.

### Annotation of glioma enhancers and their association with TFBS

To select active enhancers in glioma, we considered the presence of H3K27ac peaks in non-promoter regions and used a set of active enhancers identified previously [4]. First, we used the ChIPseeker (version 1.28.3) library's peakAnnotation function [77] to pre-filter potential H3K27ac peaks near TSS regions. The resulting set of glioma enhancers was then intersected by chromosomal coordinates with predicted TFBS in glioma cell lines using the tidygenomics R library [78] genome intersection function (version 0.1.2). Furthermore, we performed an integrative analysis of TFBS motifs in enhancers to model the relationship between each TF and distal-regulatory regions. Using the phyper function in R, we calculated probabilities based on the cumulative distribution function of the hypergeometric distribution; the probability of finding the observed number of motif instances within glioma enhancers is represented by the p-values obtained for each of the TF regulatory networks.

### DNA methylation sequencing

EZ DNA Methylation-Lightning Kit was used to bisulfite-convert DNA samples (Zymo Research, Irvine, CA, USA). SeqCap Epi CpGiant Enrichment Kit (Hoffmann-La Roche, Basel, Switzerland) probes were used to enrich each Bisulfite-Converted Sample Library in the predetermined distinct genomic regions of 80.5 Mb capture size, which included 5.6 million CpG sites on both DNA strand. The libraries were created using the “NimbleGen SeqCap Epi Library Workshop Protocol, v1.0” and “SeqCap Epi Enrichment System User's Guide, v1.2” from Hoffmann-La Roche. In brief, genomic DNA concentration was determined using a Quantus Fluorometer with QuantiFluor dsDNA System (Promega, Madison, WI, USA), and 1 g/mL streptomycin input DNA, as well as 165 pg Bisulfite-Conversion Control (viral unmethylated gDNA; SeqCap Epi Accessory Kit; Hoffmann-La Roche) were fragmented to an average size of 200 bp using the Covaris M220 (Covaris, Inc., Woburn, MA, USA). DNA fragments were tested on a 2100 Bioanalyzer using the High Sensitivity DNA Kit (Agilent Technologies, Inc., Santa Clara, CA, USA). Using the KAPA LTP Library Preparation Kit (KAPA Biosystems, Wilmington, USA), SeqCap Adapter Kit A and B (Hoffmann-La Roche), and DNA purification beads, the DNA fragments were “End-Repaired,” “A-tailed,” and the index adapters were ligated (Agencourt AMPure XP Beads; SeqCap EZ Pure Capture Bead Kit; Hoffmann-La Roche). Following that, adapter-enhanced DNA fragments were size-selected using Agencourt AMPure XP beads (SeqCap EZ Pure Capture Bead Kit) and Solid Phase Reversible Immobilization technology to exclude DNA fragments larger than 450 and smaller than 250 bp. Libraries were bisulfite transformed as described [4]. The size of the collected DNA fragments was determined using a 2100 Bioanalyzer and the High Sensitivity DNA Kit (Agilent Technologies, Inc.). Libraries were run in the Rapid Run flow cell and sequenced with paired-end sequencing (2 × 76 bp) on HiSeq 1500 (Illumina, San Diego, CA 92122 USA).

### Analysis of DNA methylation in published glioma datasets

The methylation analysis workflow was carried out using the CytoMeth tool [79], which takes Fastq files as inputs and returns the calculated DNA methylation levels (beta-values) at a base-pair level. This automated workflow includes: FastQC [64] to assess read quality, BSMAP [80] to map reads to the hg38 reference genome, Picard Tools [67] to remove PCR duplicates and methratio.py to assess coverage statistics and assign methylation levels returned as beta-values. The minimal bisulfite conversion was set to ~ 99%. The cytosines in CpG and non-CpG contexts with at least ten reads of coverage were further examined. The analysis was performed on various glioma samples: GII/GIII-IDHwt (*n* = 4), GIV (*n* = 10) and GII/GIII-IDHmut gliomas (*n* = 4). In the end, each sample yielded ~ 3.5 × 10^6^ of well-covered cytosines. However, due to DNA degeneration, the total number of cytosines shared by all samples was only ~ 350,000. The further analysis focused on differentially methylated regions rather than individual cytosines.

The DiffMeth [81] module was used to compare DNA methylation levels within promoter regions (2 kb upstream/500 bp downstream relative to TSS) as well as c-Jun motif containing regulatory regions within enhancers (50 bp long regions: 10 bp c-Jun motifs extended by flanking regions +/− 20 bp). A standard Chi2 statistical test was used to analyze statistical significance, and all groups were compared to one another. The Chi2 test compared the distribution of beta values reflecting hypo-, medium-, and hyper-methylated cytosines: [0.0–0.2], (0.2–0.6], and (0.6–1.0]; the obtained *p* values were corrected with FDR (significant if < 0.05). DiffMeth [81] was set to detect short regions of similar length to TFBS, with a median length of 22 bp. The only results that were reported were those for methylated cytosines in the CpG context.

### Analysis of DNA methylation in the TCGA dataset

CpG sites in regulatory regions of c-Jun putative target genes were intersected with promoter regions defined as 2 kb upstream/500 bp downstream relative to TSS with the annotation for the hg19 human genome [82]. GBM and LGG TCGA 450 k DNA methylation datasets were downloaded from [27]. For each defined promoter region, the median beta-values of DNA methylation were calculated per each sample. FPKM-normalized TCGA data were uploaded and Pearson correlation was calculated for selected genes for samples with matching DNA methylation and RNA-seq data. Furthermore, we searched the TCGA for information on DNA methylation in the enhancers and used the available CpG (cg02258482, cg12155676 and cg08003402).

### Survival analyses

The GlioVis web application [83] was used to conduct the analysis on the TCGA data (GBM and LGG datasets). Based on the expression of c-Jun target genes, patients were split into two subgroups (high mRNA and low mRNA levels). The association between c-Jun target expression levels and patient survival was tested using log-rank tests. For each of those genes, Kaplan–Maier plots were generated, and data from censored patients were used in the analyses.

### Immunoblotting and RT-qPCR

Whole-cell protein extracts were prepared, resolved by electrophoresis and transferred to a nitrocellulose membrane (GE Healthcare cat. number 10600003) as described [84]. After blocking with 5% non-fat milk in TBST (Tris-buffered solution pH 7.6, 0.01% Tween-20), the membranes were incubated overnight with primary antibodies diluted in TBST with 5% bovine serum albumin (BSA). The primary antibody reaction was followed by 1 h incubation with horseradish peroxidase-conjugated secondary antibodies. Immunocomplexes were detected using an enhanced chemiluminescence detection system (ECL) and Chemidoc (BioRad). The molecular weight of proteins was estimated with Cozy prestained protein ladder (High Qu GmbH cat. number PRL0102c1). Band intensities were measured by a densitometric analysis of immunoblots with BioRad Image Lab software. *P* values were calculated using GraphPad software and considered significant when *p < 0.05 (column statistics *t* test). Gene expression analysis was performed as described in [4]. Antibodies for Western Blot and the sequences of the used primers are listed in Additional file 1: Table S4.

### Isolation of nuclear extracts and electrophoretic mobility shift analysis

Nuclear extracts were prepared from cultured cells using a nuclear extraction kit: NE-PER Nuclear and Cytoplasmic Extraction Reagents (Thermo Scientific cat# 78833) according to the manufacturer instructions. Protein concentration was measured using THERMO Labsystems Multiscan EX at wavelength 570 nm with a Bradford Reagent (Sigma Life Science cat no. B6916) and a bovine serum albumin standard (Thermo Scientific cat no. 23209) was used for calibration. For EMSA, biotin-labeled and unlabeled oligonucleotides were provided by Metabion (Additional file 1: Table S4). For annealing, oligos were dissolved in water, heated to 90 °C and let to anneal for 30 min. EMSA was performed using the LightShift Chemiluminescent EMSA Kit (Thermo Scientific cat. #20148) according to the manufacturer instructions. The reactions contained: 40 fmol dsDNA, 5 µg of protein nuclear extracts and 10 mM Tris pH 7.5 buffer with 50 mM KCl, 1 mM DTT, 1.5 mM MgCl_2_, and 1.5 µg Poly (dI-dC) (in 30 μL) and all components were incubated for 30 min at room temperature and subjected to electrophoresis (70 V, 8 °C) in 6% polyacrylamide gels with 10% glycerol and Tris–borate–EDTA buffer. Then, electrophoretically separated material was transferred onto a 0.45-µm Biodyne nylon membrane (Thermo Scientific cat. # 77016) in Tris–borate–EDTA buffer and detected by chemiluminescence using a Chemidoc camera (Bio-Rad). For a competition assay, the mixture was pre-incubated with a 100-fold unlabeled probe. For a supershift assay, the protein extracts in the reaction mixture were pre-incubated for 30 min with 2 µg of anti-pS63 c-Jun antibody before adding to the DNA. Antibodies and probes used for EMSA are listed in Additional file 1: Table S4.

### Immunocytochemistry

The WG12, L0125, L0627, NTERA cells were seeded onto glass coverslip. L0125 and L0627 cells were differentiated by growing in the presence of 2% FBS. At the appropriate time cells were fixed with 4% PFA pH 7.2, washed, permeabilized with either 0.1% (cytoplasmic staining) or 0.5% (nuclear staining) Triton-X100 and blocked-in mix of 2% donkey serum and 1.5% FBS, followed by 2-h incubation with primary antibodies. Cells were then washed in PBS, incubated with corresponding Alexa Fluor A555 secondary antibodies, counterstained with DAPI (Sigma, 0.001 mg/mL, PBS) and mounted. For reagent specifications, catalog numbers and concentrations, see Additional file 1: Table S4. Images were taken using the Leica DM4000B fluorescence microscope.

### Bisulfite DNA conversion and methylation-specific polymerase chain reaction (MS-PCR)

DNA was extracted using standard phenol/chloroform methods. The purity and concentration of DNA were estimated after collecting absorbance readings at 260/280 nm. DNA (2 μg) was treated with bisulfite (EpiTect Bisulfite Kit, Qiagen, Hilden, Germany). The modified DNA was amplified using primers specific for methylated or unmethylated MGMT gene promoters, as listed in Additional file 1: Table S4. Each PCR mixture contained 1 μl of DNA, 500 nM of primers, 1 × reaction buffer containing 1.5 mM MgCl2, and 1 U HotStarTaq DNA Polymerase and 250 mM dNTPs (Promega, USA). PCR was performed with thermal conditions as follows: 95 °C for 10 min, 45 cycles of 95 °C for 30 s, 57 °C for 30 s and 72 °C for 30 s with a final extension of 72 °C for 10 min. PCR products were visualized using Agilent Tape Station system (Agilent Technologies, Palo Alto, CA, USA), yielding a band of 81 bp for a methylated product and 93 bp for an unmethylated product. Positive methylated and positive unmethylated controls (EpiTect PCR Control DNA Set Qiagen, Hilden, Germany) were included.

## Supplementary Information

**Additional file 1.** Comprises supplemental tables S1–S5 listing search results, the characterization of a grade II glioma cell line (WG12) and the antibodies and primers used in western blot and immunostaining.

**Additional file 2.** Comprises supplemental figures S1–S7 illustrating the study’s experimental and computational design, transcriptomic analyses from the TCGA between low- and grade- glioma, patient survival analyses concerning c-Jun gene targets, the DNA methylation external validation (TCGA) in c-Jun regulated genes in human gliomas, DNA methylation levels validation in Glioma Atlas concerning c-Jun motifs in putative glioma enhancers, EMSA for nuclear extracts employing probes with different methylation patterns within the *UPP1* promoter, as well as the characterization of a patient-derived primary WG12 cell line.

## Data Availability

The datasets generated and/or analyzed during the current study are available in the Synapse repository [85]. Request to access raw data is available through the European Nucleotide Archive with the accession code ERP125425 and the Gene Omnibus GSE206357. All of the scripts used to generate the computational results presented in this paper can be found in [86].

## Abbreviations

GBM: Glioblastoma
TF: Transcription factor
TCGA: The Cancer Genome Atlas
CNS: Central nervous system
WHO: World Health Organization
LGG: Lower-grade glioma
AP-1: Activator protein-1
NHA: Normal human astrocytes
GSC: Glioma stem cells
FDR: False discovery rate
FC: Fold change
PWM: Position weight matrix
TPM: Transcripts per million
GTEx: Genotype-Tissue Expression
DA: Diffuse astrocytoma
TFBS: TF binding site
TSS: Transcription start site
DEG: Differentially expressed gene
GSEA: Gene set enrichment analysis
ECM: Extracellular matrix
RPPA: Reverse protein phase assay
TAD: Topologically associated domain
PA: Pilocytic astrocytoma
HGG: High-grade glioma
EMSA: Electrophoretic mobility shift assay

## References

[1] Louis DN, Perry A, Reifenberger G, von Deimling A, Figarella-Branger D, Cavenee WK (2016). The 2016 World Health Organization classification of tumors of the central nervous system: a summary. Acta Neuropathol.

[2] Molinaro AM, Taylor JW, Wiencke JK, Wrensch MR (2019). Genetic and molecular epidemiology of adult diffuse glioma. Nat Rev Neurol.

[3] Lee TI, Young RA (2013). Transcriptional regulation and its misregulation in disease. Cell.

[4] Stępniak K, Machnicka MA, Mieczkowski J, Macioszek A, Wojtaś B, Gielniewski B (2021). Mapping chromatin accessibility and active regulatory elements reveals pathological mechanisms in human gliomas. Nat Commun.

[5] Suter DM (2020). Transcription factors and DNA play hide and seek. Trends Cell Biol.

[6] Vishnoi K, Viswakarma N, Rana A, Rana B (2020). Transcription factors in cancer development and therapy. Cancers (Basel).

[7] Grossman SR, Engreitz J, Ray JP, Nguyen TH, Hacohen N, Lander ES (2018). Positional specificity of different transcription factor classes within enhancers. Proc Natl Acad Sci U S A.

[8] Jankowski A, Szczurek E, Jauch R, Tiuryn J, Prabhakar S (2013). Comprehensive prediction in 78 human cell lines reveals rigidity and compactness of transcription factor dimers. Genome Res.

[9] Hawkins LJ, Al-attar R, Storey KB (2018). Transcriptional regulation of metabolism in disease: from transcription factors to epigenetics. PeerJ.

[10] Yu H, Li Z, Wang M (2020). Expression and prognostic role of E2F transcription factors in high-grade glioma. CNS Neurosci Ther.

[11] Thurman RE, Rynes E, Humbert R, Vierstra J, Maurano MT, Haugen E (2012). The accessible chromatin landscape of the human genome. Nature.

[12] Gazon H, Barbeau B, Mesnard JM, Peloponese JM (2018). Hijacking of the AP-1 signaling pathway during development of ATL. Front Microbiol.

[13] Hettinger K, Vikhanskaya F, Poh MK, Lee MK, de Belle I, Zhang JT (2007). c-Jun promotes cellular survival by suppression of PTEN. Cell Death Differ.

[14] Bakiri L, Lallemand D, Bossy-Wetzel E, Yaniv M (2000). Cell cycle-dependent variations in c-Jun and JunB phosphorylation: a role in the control of cyclin D1 expression. EMBO J.

[15] Piechaczyk M, Farràs R (2008). Regulation and function of JunB in cell proliferation. Biochem Soc Trans.

[16] Watanabe T, Hiasa Y, Tokumoto Y, Hirooka M, Abe M, Ikeda Y (2013). Protein kinase R modulates c-Fos and c-Jun signaling to promote proliferation of hepatocellular carcinoma with hepatitis C virus infection. PLoS ONE.

[17] Zenz R, Eferl R, Scheinecker C, Redlich K, Smolen J, Schonthaler HB (2008). Activator protein 1 (Fos/Jun) functions in inflammatory bone and skin disease. Arthritis Res Ther.

[18] Santaguida M, Schepers K, King B, Sabnis AJ, Forsberg EC, Attema JL (2009). JunB protects against myeloid malignancies by limiting hematopoietic stem cell proliferation and differentiation without affecting self-renewal. Cancer Cell.

[19] Regulomics. http://regulomics.mimuw.edu.pl/GliomaAtlas/

[20] Kulakovskiy IV, Vorontsov IE, Yevshin IS, Sharipov RN, Fedorova AD, Rumynskiy EI (2018). HOCOMOCO: towards a complete collection of transcription factor binding models for human and mouse via large-scale ChIP-Seq analysis. Nucleic Acids Res.

[21] Seifert M, Garbe M, Friedrich B, Mittelbronn M, Klink B (2015). Comparative transcriptomics reveals similarities and differences between astrocytoma grades. BMC Cancer.

[22] Xu B (2021). Prediction and analysis of hub genes between glioblastoma and low-grade glioma using bioinformatics analysis. Medicine (Baltimore).

[23] Karunasena E, McIver LJ, Rood BR, Wu X, Zhu H, Bavarva JH (2014). Somatic intronic microsatellite loci differentiate glioblastoma from lower-grade gliomas. Oncotarget.

[24] Bhatlekar S, Fields JZ, Boman BM (2014). HOX genes and their role in the development of human cancers. J Mol Med.

[25] Guo YB, Shao YM, Chen J, Xu SB, Zhang XD, Wang MR (2016). Effect of overexpression of HOX genes on its invasive tendency in cerebral glioma. Oncol Lett.

[26] Cain B, Gebelein B (2021). Mechanisms underlying hox-mediated transcriptional outcomes. Front Cell Dev Biol.

[27] BroadInstitute Firehose. https://gdac.broadinstitute.org

[28] Zhao J, Zhang L, Dong X, Liu L, Huo L, Chen H (2018). High expression of vimentin is associated with progression and a poor outcome in glioblastoma. Appl Immunohistochem Mol Morphol.

[29] Zhu QS, Rosenblatt K, Huang KL, Lahat G, Brobey R, Bolshakov S (2011). Vimentin is a novel AKT1 target mediating motility and invasion. Oncogene.

[30] Angel P, Hattori K, Smeal T, Karin M (1988). The jun proto-oncogene is positively autoregulated by its product, Jun/AP-1. Cell.

[31] Detilleux D, Spill YG, Balaramane D, Weber M, Bardet AF (2022). Pan-cancer predictions of transcription factors mediating aberrant DNA methylation. Epigenet Chromatin.

[32] Lioznova AV, Khamis AM, Artemov AV, Besedina E, Ramensky V, Bajic VB (2019). CpG traffic lights are markers of regulatory regions in human genome. BMC Genomics.

[33] Héberlé É, Bardet AF (2019). Sensitivity of transcription factors to DNA methylation. Essays Biochem.

[34] Malta TM, De Souza CF, Sabedot TS, Silva TC, Mosella MS, Kalkanis SN (2018). Glioma CpG island methylator phenotype (G-CIMP): biological and clinical implications. Neuro Oncol.

[35] de Souza CF, Sabedot TS, Malta TM, Stetson L, Morozova O, Sokolov A (2018). A distinct DNA methylation shift in a subset of glioma CpG island methylator phenotypes during tumor recurrence. Cell Rep.

[36] Shinawi T, Hill VK, Krex D, Schackert G, Gentle D, Morris MR (2013). DNA methylation profiles of long- and short-term glioblastoma survivors. Epigenetics.

[37] Kolat D, Kaluzinska Z, Bednarek AK, Pluciennik E (2019). The biological characteristics of transcription factors AP-2α and AP-2γ and their importance in various types of cancers. Biosci Rep.

[38] Stuart ET, Kioussi C, Gruss P, Aguzzi A (1995). PAX5 expression correlates with increasing malignancy in human astrocytomas. Clin Cancer Res.

[39] Rhie SK, Yao L, Luo Z, Witt H, Schreiner S, Guo Y (2018). ZFX acts as a transcriptional activator in multiple types of human tumors by binding downstream from transcription start sites at the majority of CpG island promoters. Genome Res.

[40] Fang X, Huang Z, Zhou W, Wu Q, Sloan AE, Ouyang G (2014). The zinc finger transcription factor ZFX Is required for maintaining the tumorigenic potential of glioblastoma stem cells. Stem Cells.

[41] Vleugel MM, Greijer AE, Bos R, van der Wall E, van Diest PJ (2006). c-Jun activation is associated with proliferation and angiogenesis in invasive breast cancer. Hum Pathol.

[42] Brennan A, Leech JT, Kad NM, Mason JM (2020). Selective antagonism of cJun for cancer therapy. J Exp Clin Cancer Res.

[43] Wu S, Du Y, Beckford J, Alachkar H (2018). Upregulation of the EMT marker vimentin is associated with poor clinical outcome in acute myeloid leukemia. J Transl Med.

[44] Meyer-Schaller N, Cardner M, Diepenbruck M, Saxena M, Tiede S, Lüönd F (2019). A hierarchical regulatory landscape during the multiple stages of EMT. Dev Cell.

[45] Shi Y, Ping YF, Zhou W, He ZC, Chen C, Bian BSJ (2017). Tumour-associated macrophages secrete pleiotrophin to promote PTPRZ1 signalling in glioblastoma stem cells for tumour growth. Nat Commun.

[46] Meng F, Li W, Li C, Gao Z, Guo K, Song S (2015). CCL18 promotes epithelial-mesenchymal transition, invasion and migration of pancreatic cancer cells in pancreatic ductal adenocarcinoma. Int J Oncol.

[47] Kim H, Claps G, Möller A, Bowtell D, Lu X, Ronai ZA (2014). Siah2 regulates tight junction integrity and cell polarity through control of ASPP2 stability. Oncogene.

[48] Chen A, Wong CSF, Liu MCP, House CM, Sceneay J, Bowtell DD (2015). The ubiquitin ligase Siah is a novel regulator of Zeb1 in breast cancer. Oncotarget.

[49] Guan Y, Bhandari A, Zhang X, Wang O (2019). Uridine phosphorylase 1 associates to biological and clinical significance in thyroid carcinoma cell lines. J Cell Mol Med.

[50] Naz S, Bashir M, Ranganathan P, Bodapati P, Santosh V, Kondaiah P (2014). Protumorigenic actions of S100A2 involve regulation of PI3/Akt signaling and functional interaction with Smad3. Carcinogenesis.

[51] Shaulian E, Karin M (2001). AP-1 in cell proliferation and survival. Oncogene.

[52] Rodríguez-Martínez JA, Reinke AW, Bhimsaria D, Keating AE, Ansari AZ (2017). Combinatorial bZIP dimers display complex DNA-binding specificity landscapes. Elife.

[53] Elias MC, Tozer KR, Silber JR, Mikheeva S, Deng M, Morrison RS (2005). TWIST is expressed in human gliomas and promotes invasion. Neoplasia.

[54] Kurscheid S, Bady P, Sciuscio D, Samarzija I, Shay T, Vassallo I (2015). Chromosome 7 gain and DNA hypermethylation at the HOXA10 locus are associated with expression of a stem cell related HOX-signature in glioblastoma. Genome Biol.

[55] Wu Y, Zhang X, Zehner ZE (2003). c-Jun and the dominant-negative mutant, TAM67, induce vimentin gene expression by interacting with the activator Sp1. Oncogene.

[56] Bell O, Tiwari VK, Thomä NH, Schübeler D (2011). Determinants and dynamics of genome accessibility. Nat Rev Genet.

[57] Król SK, Kaczmarczyk A, Wojnicki K, Wojtas B, Gielniewski B, Grajkowska W (2020). Aberrantly expressed recql4 helicase supports proliferation and drug resistance of human glioma cells and glioma stem cells. Cancers (Basel).

[58] Ciechomska IA, Wojnick K, Wojtas B, Szadkowska P, Poleszak K, Kaza B, et al. In search for reliable markers of glioma-induced polarization of microglia. Exploring responses of glioblastoma patient-derived cell cultures to drugs reveals new therapeutic opportunities. Preprints 2023, 2023010585. 10.20944/preprints202301.0585.v1.

[59] Galli R, Binda E, Orfanelli U, Cipelletti B, Gritti A, De Vitis S (2004). Isolation and characterization of tumorigenic, stem-like neural precursors from human glioblastoma. Cancer Res.

[60] Di Tomaso T, Mazzoleni S, Wang E, Sovena G, Clavenna D, Franzin A (2010). Immunobiological characterization of cancer stem cells isolated from glioblastoma patients. Clin Cancer Res.

[61] Mazzoleni S, Politi LS, Pala M, Cominelli M, Franzin A, Sergi LS (2010). Epidermal growth factor receptor expression identifies functionally and molecularly distinct tumor-initiating cells in human glioblastoma multiforme and is required for gliomagenesis. Cancer Res.

[62] Ciechomska IA, Przanowski P, Jackl J, Wojtas B, Kaminska B (2016). BIX01294, an inhibitor of histone methyltransferase, induces autophagy-dependent differentiation of glioma stem-like cells. Sci Rep.

[63] Ciechomska IA, Gielniewski B, Wojtas B, Kaminska B, Mieczkowski J (2020). EGFR/FOXO3a/BIM signaling pathway determines chemosensitivity of BMP4-differentiated glioma stem cells to temozolomide. Exp Mol Med.

[64] Andrews S. FastQC—a quality control tool for high throughput sequence data. Babraham Bioinforma. 2010. http://www.bioinformatics.babraham.ac.uk/projects/fastqc/.

[65] Gordon A, Hannon GJ. Fastx-toolkit. FASTQ/A short-reads pre-processing tools. 2010. http://hannonlab.cshl.edu/fastx_toolkit.

[66] Langmead B, Salzberg SL (2012). Fast gapped-read alignment with Bowtie 2. Nat Methods.

[67] Broad Institute. Picard tools—by broad institute. Github. 2009.

[68] Cunningham F, Achuthan P, Akanni W, Allen J, Amode MR, Armean IM, et al. Ensembl 2019. 2019;47(November 2018):745–51.

[69] Love MI, Huber W, Anders S (2014). Moderated estimation of fold change and dispersion for RNA-seq data with DESeq2. Genome Biol.

[70] Yu G, Wang LG, Han Y, He QY (2012). ClusterProfiler: an R package for comparing biological themes among gene clusters. OMICS: J Integr Biol.

[71] Grant CE, Bailey TL, Noble WS (2011). FIMO: scanning for occurrences of a given motif. Bioinformatics.

[72] D’Oliveira Albanus R, Kyono Y, Hensley J, Varshney A, Orchard P, Kitzman JO (2021). Chromatin information content landscapes inform transcription factor and DNA interactions. Nat Commun.

[73] Bolger AM, Lohse M, Usadel B (2014). Trimmomatic: a flexible trimmer for Illumina sequence data. Bioinformatics.

[74] Dobin A, Davis CA, Schlesinger F, Drenkow J, Zaleski C, Jha S (2013). STAR: ultrafast universal RNA-seq aligner. Bioinformatics.

[75] Liao Y, Smyth GK, Shi W (2014). FeatureCounts: an efficient general purpose program for assigning sequence reads to genomic features. Bioinformatics.

[76] Tang Z, Kang B, Li C, Chen T, Zhang Z (2019). GEPIA2: an enhanced web server for large-scale expression profiling and interactive analysis. Nucleic Acids Res.

[77] Yu G, Wang LG, He QY (2015). ChIP seeker: an R/bioconductor package for ChIP peak annotation, comparison and visualization. Bioinformatics.

[78] tidygenomics. https://github.com/const-ae/tidygenomics.

[79] CytoMeth. https://github.com/mdraminski/CytoMeth.

[80] Xi Y, Li W (2009). BSMAP: whole genome bisulfite sequence MAPping program. BMC Bioinform.

[81] Akalin A, Kormaksson M, Li S, Garrett-Bakelman FE, Figueroa ME, Melnick A (2012). MethylKit: a comprehensive R package for the analysis of genome-wide DNA methylation profiles. Genome Biol.

[82] Flicek P, Amode MR, Barrell D, Beal K, Billis K, Brent S, et al. Ensembl 2014. 2014;42(December 2013):749–55.

[83] GlioVis. http://gliovis.bioinfo.cnio.es.

[84] Ciechomska I, Pyrzynska B, Kazmierczak P, Kaminska B (2003). Inhibition of Akt kinase signalling and activation of Forkhead are indispensable for upregulation of FasL expression in apoptosis of glioma cells. Oncogene.

[85] Roura AJ. Synapse repository. www.synapse.org/TFBSglioma.

[86] Roura AJ. GitHub. https://github.com/ajroura22/glioma_TFBS.

